# Evaluation of flood metrics across the Mississippi-Atchafalaya River Basin and their relation to flood damages

**DOI:** 10.1371/journal.pone.0307486

**Published:** 2024-10-09

**Authors:** Keith E. Schilling, Elliot S. Anderson, Jerry Mount, Kelly Suttles, Philip W. Gassman, Natalja Cerkasova, Michael J. White, Jeffrey G. Arnold

**Affiliations:** 1 Iowa Geological Survey, University of Iowa, Iowa City, Iowa, United States of America; 2 IIHR-Hydroscience & Engineering, University of Iowa, Iowa City, Iowa, United States of America; 3 Environmental Defense Fund, Raleigh, North Carolina, United States of America; 4 Center for Rural and Agricultural Development, Iowa State University, Ames, Iowa, United States of America; 5 Texas A&M AgriLife Research, Blackland Research and Extension Center, Temple, Texas, United States of America; 6 Department of Agriculture, Grassland Soil and Water Research Laboratory, Agricultural Research Service, U.S. Temple, Texas, United States of America; University 20 Aout 1955 skikda, Algeria, ALGERIA

## Abstract

Societal risks from flooding are evident at a range of spatial scales and climate change will exacerbate these risks in the future. Assessing flood risks across broad geographical regions is a challenge, and often done using streamflow time-series records or hydrologic models. In this study, we used a national-scale hydrological model to identify, assess, and map 16 different streamflow metrics that could be used to describe flood risks across 34,987 HUC12 subwatersheds within the Mississippi-Atchafalaya River Basin (MARB). A clear spatial difference was observed among two different classes of metrics. Watersheds in the eastern half of the MARB exhibited higher overall flows as characterized by the mean, median, and maximum daily values, whereas western MARB watersheds were associated with flood indicative of high extreme flows such as skewness, standardized streamflow index and top days. Total agricultural and building losses within HUC12 watersheds were related to flood metrics and those focused on higher overall flows were more correlated to expected annual losses (EAL) than extreme value metrics. Results from this study are useful for identifying continental scale patterns of flood risks within the MARB and should be considered a launching point from which to improve the connections between watershed scale risks and the potential use of natural infrastructure practices to reduce these risks.

## Introduction

Societal risks from flooding are evident at a global scale [e.g., 1–3], with floods causing human fatalities and massive economic losses [4, 5]. In many regions, the frequency of major flood events is projected to increase with future climate change [6–8]. Assessing flood risks is often done using historical analyses of streamflow time-series records [6, 9, 10] or using hydrological modeling and climate projections to forecast flood risks [e.g., 11, 12]. Despite ongoing research, reliable estimation of flood hazards and flood risks remains a challenge [11, 13].

Assessment of flood risks often involves one of two approaches: the annual maximum flood (AMF) or the peaks over threshold (POT) [14, 15]. The AMF method utilizes the maximum streamflow value in a given year and has the advantage of simplicity and independence of extreme values [16]. A disadvantage of this method is that the record may contain non-extreme values from dry years or miss other flood peaks occurring in the same calendar year [15]. The POT method provides a larger sample size and captures all flood peaks above a pre-determined threshold but is sensitive to the selection of an appropriate threshold level and assuring that flood peaks are independent. In some cases, multiple threshold levels are evaluated from the same streamflow record [6], or threshold values are extracted from site-specific stage measurements [17]. POT methods have been increasingly used in flood studies around the world [e.g., 8, 18–20]. Other studies of flood risk have focused on specific statistical features in streamflow records, including flash floods [21] and so-called “strange floods” that describe the upper tail of a hydrograph defined by the flood peak divided by the sample 10-year flood magnitude [22].

The vast majority of flood-related studies rely on measured discharge records from streamflow gauges to evaluate flood risks, but most rivers and streams are ungauged. For example, in the Mississippi-Atchafalaya River Basin (MARB) in the United States, which drains more than 3.2 million km^2^ there are approximately 2,850 gauging stations. In contrast, there are nearly 35,000 small subwatersheds within the MARB [23], indicating that less than 10% are likely to be gauged. Hence, there exists a large subset of research devoted to making flood predictions in ungauged basins [e.g., 24, 25]. Streamflow predictions in ungauged basins are often based on hydrological models and parameters regionalized from measured data collected nearby [26].

Methods using either gauged streamflow records or simulated hydrologic data have proven effective at quantifying flood risk within a basin [27], as these techniques estimate exceedance probabilities corresponding to a particular gauge’s flow values. However, hydrologic variations among watersheds complicate the ability to assess flood risk at multiple locations. Investigating flood risk spatially may identify watersheds where societal damages due to flooding are disproportionately high [28], which can help prioritize areas where flood mitigation efforts will have the greatest benefit [29]. Many studies have analyzed economic and demographic data alongside inundation mapping to identify at-risk areas [28, 30, 31]. Others have taken a purely hydrologic approach—using various hydrologic parameters as a proxy for risk [32, 33]. Given the ubiquity of observed and model hydrologic data, spatial assessments of risk that solely utilize hydrology are easier to apply at continental scales [34]. Still, it can be unclear how parameters from hydrologic time-series relate to flooding [35].

In this study, we used a national-scale hydrological model to identify and map potential streamflow metrics that could be used to describe flood risks across ~35,000 ungauged subwatersheds present within the MARB. With widely variable climate, topography, and land use across the basin, we show that the selection of different flood metrics reveals different spatial patterns of flooding risks. From the potential streamflow flood metrics evaluated, we further identify metrics that are most correlated to actual reported flood damages to property and agricultural lands. This study fits within a larger overall project focused on identifying and prioritizing flood risks in subwatersheds and regions of the MARB where future natural infrastructure (NI) practices could be installed for flood risk reductions [23].

## Methods and materials

### MARB description

**Fig 1:**
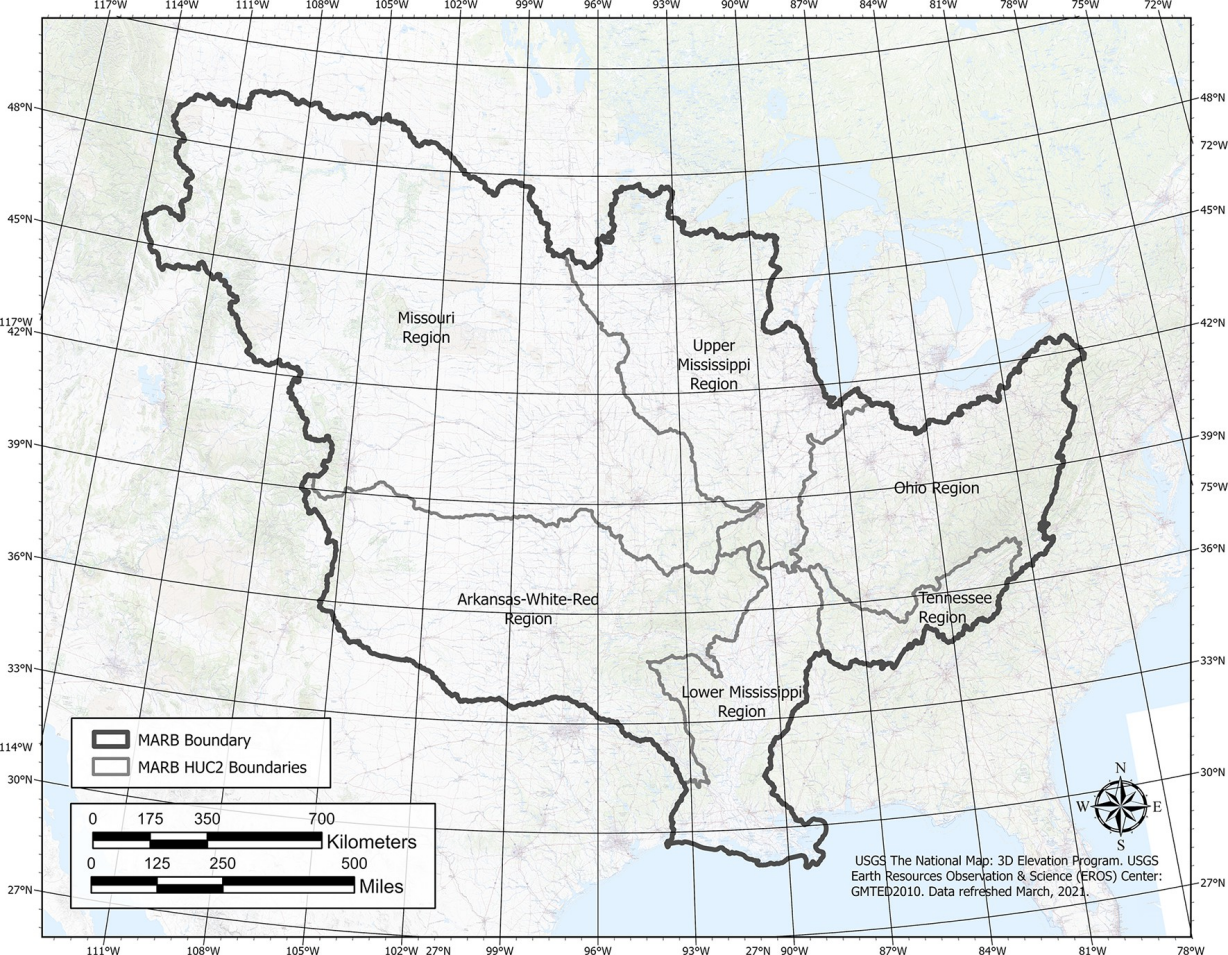
Extent of the Mississippi-Atchafalaya basin within the U.S.

The MARB, extending east to west from the Appalachian Mountains in New York to the Rocky Mountains in western Montana and north to south from Canada to the Gulf of Mexico, drains an area of 3,208,700 km^2^ or about 41% of the conterminous United States (Fig 1). The MARB drainage area includes parts or all of 31 states and portions of two Canadian provinces. In a basin this large, climate, topography, and land use vary widely across the region (see Schilling et al. [23]). Long-term annual precipitation (P) increases from the drier High Plains region (<500 mm) to the southeast U.S., where annual P exceeds 1,500 mm. Land slopes are commonly less than 2% in the recently glaciated Midwest, Mississippi River delta region, and High Plains but exceed 7% in older glacial landscapes, Driftless region of Wisconsin, and in mountainous regions that include the Appalachians, Rock Mountains, and Central Uplift zone in southern Missouri and Arkansas. The dominant land uses in the MARB include forest (32%), grassland/pasture/hay (30%), all cropland (25%), and urban (5%), with less amounts of wetland, fallow/barren and water (combined <8%). Row crops of corn and soybeans in rotation are intensively grown in the expansive Corn Belt region that stretches from the eastern Missouri River Basin across the Upper Mississippi River Basin to the western part of the Ohio River Basin. Additional information about land use, conservation practices and other MARB characteristics are provided in [36].

MARB climate conditions reflect a considerable range representative of the continental-scale size of the Mississippi River system. Mean monthly winter temperatures range from −12°C in subarctic northern Minnesota to 13°C in subtropical southern Louisiana; the corresponding mean monthly summer temperatures span from 21°C in Minnesota to 28°C in Louisiana (Kesel and Severin, 2024). The lowest average monthly precipitation (2.5 cm) occurs in the western part of the MARB (western and northern Great Plains region) and increases to 7.5 cm across much of the eastern MARB region (Ohio River Basin) and to 13 cm or more in the southern MARB subregion [37]. The MARB can also be generally categorized into four primary climate regions based on the Köppen-Geiger system [38] as follows: (1) Ari-Steppe-Cold (Bsk) in the western MARB (western and northern Great Plains region), (2) Cold-Without dry season-Warm season (Dfb) in the far northern part of the MARB (northern Minnesota and Wisconsin), (3) Cold-Without dry season-Warm season (Dfa) across much of the MARB Corn Belt subregion), and (4) Temperate-Without dry season-Warm season (Cfa) that encompasses most of the southern part of the MARB.

### SWAT+ model

The Soil and Water Assessment Tool Plus (SWAT+) ecohydrological model was used to simulate the hydrologic balance and resulting streamflows for the MARB system, which is based on predecessor SWAT codes [39–43]. The SWAT/SWAT+ models together represent extensive algorithm evolution over a period of three decades [43–46], and have been applied worldwide for a broad suite of water resource problems and environmental conditions for study regions that range from field-scale drainage areas to transnational river systems [41, 46–53]. SWAT applications have also been performed for all of the major MARB water resource regions as noted by Schilling et al. [23].

The SWAT+ simulations incorporated in this study have been performed within the National Agroecosystem Model (NAM), which provides the capability of executing the model for different areal resolutions including specific crop fields [54–57]. The utilization of SWAT+ within NAM provides enhanced flexibility in depicting various watershed elements (e.g., rivers, ponds, wetlands, rice paddies, crop fields, urban areas), and the hydrologic and pollutant flows between watershed elements. The NAM system is currently being calibrated and refined for the entire contiguous U.S., using a structure that consists of 2,121 individual SWAT+ watershed simulations [57]. These watersheds coincide with the boundaries of 8-digit watershed hydrologic unit codes (HUCs), which are part of the hierarchical system of basins and watersheds that have been delineated by collaborating federal agencies [23, 58]. The NAM structure also accommodates other HUC levels, such as providing output for the 86,016 12-digit watersheds across the contiguous U.S. [57]. The NAM MARB structure is comprised of 845 8-digit (HUC8s) and 34,944 12-digit watersheds (HUC12s) [36].

The smallest computation units incorporated in NAM are represented by hydrologic response units (HRUs) in SWAT+, which currently consist of roughly 4.6 million agricultural fields and 2.3 million non-agricultural areas [57]. SWAT+ is executed for each of these agricultural and non-agricultural HRUs, which respectively average 24 ha and 323 ha in size [57]. A complete set of management practices, such as planting, tillage, fertilizing, grazing, harvesting and irrigating, along with conservation practices and subsurface tile drainage, are accounted for the agricultural HRUs as applicable. However, output is generated at the 12-digit watershed level at present rather than for specific HRUs.

**Fig 2:**
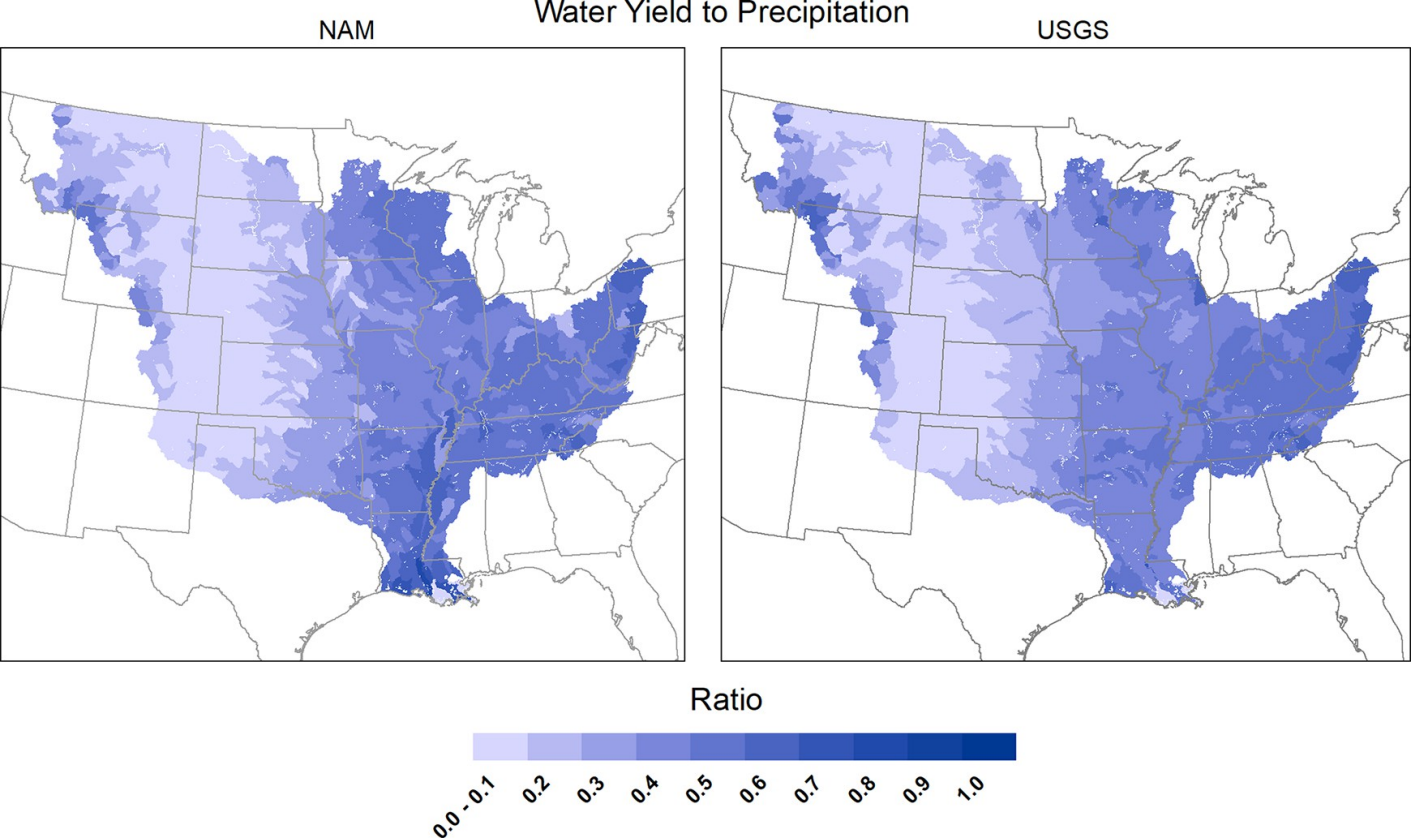
Comparison of discharge-precipitation ratios measured at USGS stream gauging sites with ratios determined with SWAT+ simulations.

Intensive testing of SWAT+ within NAM is being conducted in two primary phases for the MARB region and the broader continental United States (CONUS). The first phase is focused on evaluating model components on a “soft data” basis, which consists of establishing the ability of SWAT+ to realistically replicate fundamental hydrologic characteristics for different stream systems (e.g., surface flow versus baseflow, subsurface tile drainage flow, groundwater effects), crop growth and yields, and other known attributes of specific watersheds. Calibration and validation of SWAT+ predicted corn and soybean yields has been reported for the entire CONUS [56], and similar testing is ongoing for other crops such as wheat and rice. Execution of different groundwater functions, including groundwater pumping, tile drainage, and groundwater-surface water interactions, has been performed with the SWAT+ gwflow component for six 8-digit watersheds representative of different U.S. regions [55]. The model’s ability to represent overall water balance, including discharge to precipitation ratios, has been confirmed for the MARB (Fig 2) and other CONUS regions. This is the stage of SWAT+ testing within NAM that represents the streamflow data used in this study.

The second phase of NAM testing has been initiated which consists of calibrating flow duration curves (FDCs) and estimating sediment and nutrient loads across CONUS. Reliable results have been obtained with comparing FDCs versus the water balance that was obtained on the basis of the soft calibration process. A more rigorous testing step currently involves comparing FDCs with USGS gauge sites representative of major stream systems. Average annual suspended sediment, total nitrogen and total phosphorus loads estimated with SWAT+ are also being compared with corresponding loads determined with Load Estimator (LOADEST; https://water.usgs.gov/software/loadest/) at USGS gauges throughout CONUS. It is stressed that the testing of SWAT+ within NAM is an evolving process that incorporates refinements as improved monitoring and other data becomes available.

### Definition of streamflow metrics

The calibrated SWAT models simulated streamflow at each HUC12 subwatershed from January 1, 2000, to December 31, 2018—a period of 19 years (6,940 days). The model outputs included daily estimates of the flow’s runoff, groundwater, and subsurface drainage components, which were summed to obtain total streamflow for each HUC12. Flow values were normalized by their tributary area to account for the variability in subwatershed size. Thus, modeled time-series of streamflow (mm/day) were generated in all ~35,000 HUC12s within the MARB. We calculated several statistics for each time-series, most of which are regularly used in hydrologic applications. These metrics were identified as potentially being informative of a basin’s flood risk, thus warranting exploration throughout the MARB. They are described herein and summarized in S1 Table.

The first group contained standard parametric statistics. The arithmetic mean is the average daily streamflow in a HUC12 and is defined as

$$mean=\frac{1}{6940}\sum_{i=1/1/2000}^{12/31/2018}Q_{i}$$

where Q_i_ is the daily streamflow on day i and 6940 is the number of days in our 19-year simulated timeframe. The arithmetic mean describes the watershed’s overall water volume and can be interpreted as a measure of relative wetness. The standard deviation describes the dispersion in the daily flow values. It is defined as

$$std=\sqrt{\frac{\sum_{i=1/1/2000}^{12/31/2018}{\left(Q_{i}-mean\right)}^{2}}{6940}}$$

Skewness quantifies the asymmetry of flow values. Daily streamflow in free-flowing rivers throughout the MARB consistently exhibits positive skewness [59], where high-flow events are often orders of magnitude larger than low-flow conditions. Skewness is defined as

$$skew=\frac{\sum_{i=1/1/2000}^{12/31/2018}{\left(Q_{i}-mean\right)}^{3}}{(6940-1)*{std}^{3}}$$

Several standard nonparametric statistics were also found, including the 50^th^ (median), 90^th^, and 99^th^ percentiles and the maximum daily flow.

Hydrologists and geomorphologists have identified parameters that describe the typical flow conditions under which streams exceed their bankfull capacity [60–62]. The ratio between the 99^th^ percentile and the mean daily flow has been used to approximate bankfull flow thresholds at sites with daily hydrologic observations but limited geomorphologic data [63, 64]. This statistic (hereafter referred to as ratio) provided a way to quantify bankfull discharge throughout the MARB. In this study, it was defined as

$$ratio=\frac{99\%}{mean}$$

Stream flashiness is a hydrologic characteristic that describes the day-to-day volatility of streamflow values. The most common metric for flashiness is the Richards-Baker flashiness index (RB; [65]). For a given timeframe, the RB statistic calculates the daily changes in streamflow and normalizes this by the river’s total discharge. It is formally defined as

$$RB=\frac{\sum_{i=1/1/2000}^{12/31/2018}\left|Q_{i}-Q_{i-1}\right|}{\sum_{i=1/1/2000}^{12/31/2018}Q_{i}}$$

The standardized streamflow index (SSI) is often used to describe anomalous streamflow conditions [66, 67]. It quantifies the number of standard deviations from the mean for a given flow value. While its typical application is for drought assessment [68], the SSI can also assess abnormally wet periods [69]. The SSI was calculated on a daily basis in all HUC12s. SSI calculations require that that time-series are transformed into a normal distribution [70]. Lognormal distributions can adequately model daily streamflow in the MARB [59], so each SWAT flow output was log-transformed to normalize the datasets. Days with 0.0 mm of streamflow were set to 0.01 mm prior to transformation—a value corresponding to the minimum nonzero modeled flow. The daily SSI values were calculated using the following equation

$$SSI=\frac{q_{i}-\bar{q}}{\sigma }$$

where q_i_ is the log-transformed value on day i, $\bar{q}$ is the mean of the log-transformed flows, and σ is the standard deviation of the log-transformed flows. For each HUC12, we found the maximum SSI value (maxSSI) and number of days where SSI was greater than 3 (SSI_3; i.e., the number of days the flow was more than 3 standard deviations above the mean).

Finally, we created an additional statistic that quantified the percentage of discharge occurring in the wettest days. A high proportion of flow discharged during only a few days of record indicates a watershed prone to intermittent high flow events and potential flooding. We termed this metric Top Days (TD) and defined it as

$$TD(k)=\frac{\sum_{1}^{k}{max}_{k}(Q)}{\sum_{i=1/1/2000}^{12/31/2018}Q_{i}}$$

where k is the number of maximum flow days to include in the calculation, e.g., for k = 4, the TD metric sums the flows from 4 wettest days and divides this by the basin’s total flow. Therefore, TD is always a percentage bounded between 0.0 and 1.0. TD metrics were calculated using k values of 1, 4, 21, 69, and 139, corresponding to the 100, 99.9, 99.5, 99, and 98 flow percentiles over the record of 6940 days.

Each of these statistics can be calculated by utilizing daily streamflow records. They characterize various hydrologic regimes in all of the HUC12s that may help prioritize flood risk. It was unknown whether these parameters exhibit similar spatial tendencies—e.g. if watersheds with large maximum flows also contained the highest skewness values. In such an instance, using both the max and skew metrics to prioritize watersheds would prove redundant, as much of the same hydrologic behavior is being captured. To investigate the potential of informational redundancy among the metrics, we calculated their correlations and created spatial maps that color-coded the values by watershed. The statistics for each HUC12 are included in the S1 File.

### Flood damages

Flood events are caused by many factors that can be classified into hydroclimatic, hydrological and hydrograph-based categories for relation to flood damages [71]. Hydroclimatic differences typically arise based on regional to subcontinental scale meteorological and climate patterns, whereas hydrological differences relate to hydrometeorological variables (rainfall, snowmelt, etc.) occurring at the watershed scale. Hydrograph-based events are typically tailored to describe localized flood generation processes [71, 72]. In this study, we focused on evaluating the relation of various streamflow metrics to flood damages rather than on specific flood causes. The streamflow metrics considered herein include those linked to regional climatological as well as watershed-scale and localized hydrologic causes. The severity of floods can be described based on recurrence intervals (e.g., extreme floods with a return period >100 years; [73, 74]), but this methodology does not provide other critical information such as flood duration and extent of inundation [75]. Flood damages arising from various causes and ranges of severity include damages caused by physical contact with water and those occurring outside the flood event over longer time scales [76]. Although direct flood damages are more quantifiable, flood damage assessments often depend on simplified approaches and include many assumptions [76].

Flood damage data were not available for individual HUC12 watersheds, but estimated annual losses for agriculture and buildings damages are reported by census tract in the National Risk Index (NRI) [77]. To convert the census tract resolution to a watershed basis we assume damages reported within the census tracts directly correlate with flooding incidents that affected both buildings and agricultural lands situated within identifiable floodplains in those tracts.

The raster floodplain data from Samela et al. [78] was first resampled from the original 90 m to a 10 m cell resolution. Similarly, the census tract polygons for building footprints for each tract within the NRI database were rasterized though into 10 m cells and those located within the floodplains were selected. Building cells within the floodplain were counted and assigned an Estimated Annual Loss for Buildings (EALB) value by dividing the total EALB value for the tract by the number of cells within the floodplain. For agricultural losses, the National Land Cover Database (NLCD) [79] data were resampled to 10 m and clipped by each Census tract. Agricultural cells were selected and those within each tract were counted and assigned a value for Estimated Annual Loss–Agriculture (EALA) created by dividing the total EALA for a tract by the number of cells. The EALA and EALB rasters were then mosaicked into a single raster for each HUC12 watershed in the MARB.

## Results

### Population statistics of streamflow metrics

Daily streamflow values estimated at ~35,000 HUC12s over a period of 19 years showed clear variations as would be expected for a basin as large as the MARB (Table 1). For example, mean daily flows averaged 0.54 mm among the HUC12 watersheds and had median and maximum values of 0.50 and 3.3 mm. Maximum daily flows averaged 32.3 mm, with median and maximum values of 30.3 and 167 mm (Table 1). Dimensionless values such as the ratio and skewness did not vary as much or as systematically as daily flows, but maximum values for these metrics were still much higher than the overall populations. The RB index—also dimensionless—had the smallest range (0.0–1.9) and was the one metric without substantially higher maximum values. TD metrics indicating the percentage of the total flow record contained within the various time windows increased with a larger number of days included. The mean percentage of total flow occurring within the 1-day and 139-day periods increased from 1.2% to 41% among all the HUC12 watersheds, with maximum percentages of 11.5% and 100%, respectively (Table 1).

**Table 1:** Descriptive statistics for flood metrics calculated for all HUC12 subwatersheds within the MARB.

stat	mean	std	50%	90%	99%	max	ratio	skew	RB	TD_1	TD_4	TD_21	TD_69	TD_139	maxSSI	SSI_3
mean	0.54	1.57	0.08	1.31	7.44	32.28	16.38	8.72	0.73	1.21%	3.83%	13.22%	28.31%	41.25%	3.10	26.6
std	0.41	0.92	0.13	1.14	4.59	20.35	4.99	3.55	0.18	0.79%	2.21%	6.52%	11.77%	14.58%	0.78	55.9
10%	0.09	0.43	0.00	0.08	1.85	9.94	10.85	5.27	0.50	0.51%	1.79%	6.89%	16.34%	25.74%	2.34	0.0
25%	0.16	0.67	0.01	0.25	3.07	14.64	12.75	6.34	0.62	0.68%	2.30%	8.50%	19.62%	30.46%	2.55	0.0
50%	0.50	1.64	0.04	1.13	7.76	30.35	15.31	8.06	0.74	0.98%	3.20%	11.29%	24.93%	37.47%	2.91	0.0
75%	0.81	2.24	0.10	2.08	10.70	44.33	19.56	10.25	0.86	1.51%	4.84%	16.79%	35.46%	50.52%	3.48	18.0
90%	1.08	2.68	0.20	2.89	12.95	58.35	24.15	12.69	0.96	2.14%	6.67%	22.39%	45.75%	63.44%	4.12	108.0
99%	1.65	3.94	0.59	4.49	19.76	94.48	28.22	21.03	1.14	4.19%	11.83%	34.51%	61.61%	78.58%	5.62	240.0
max	3.30	6.99	1.71	8.59	36.24	167.45	56.89	54.95	1.69	11.55%	23.96%	52.76%	82.20%	100.00%	40.71	308.0

**Fig 3:**
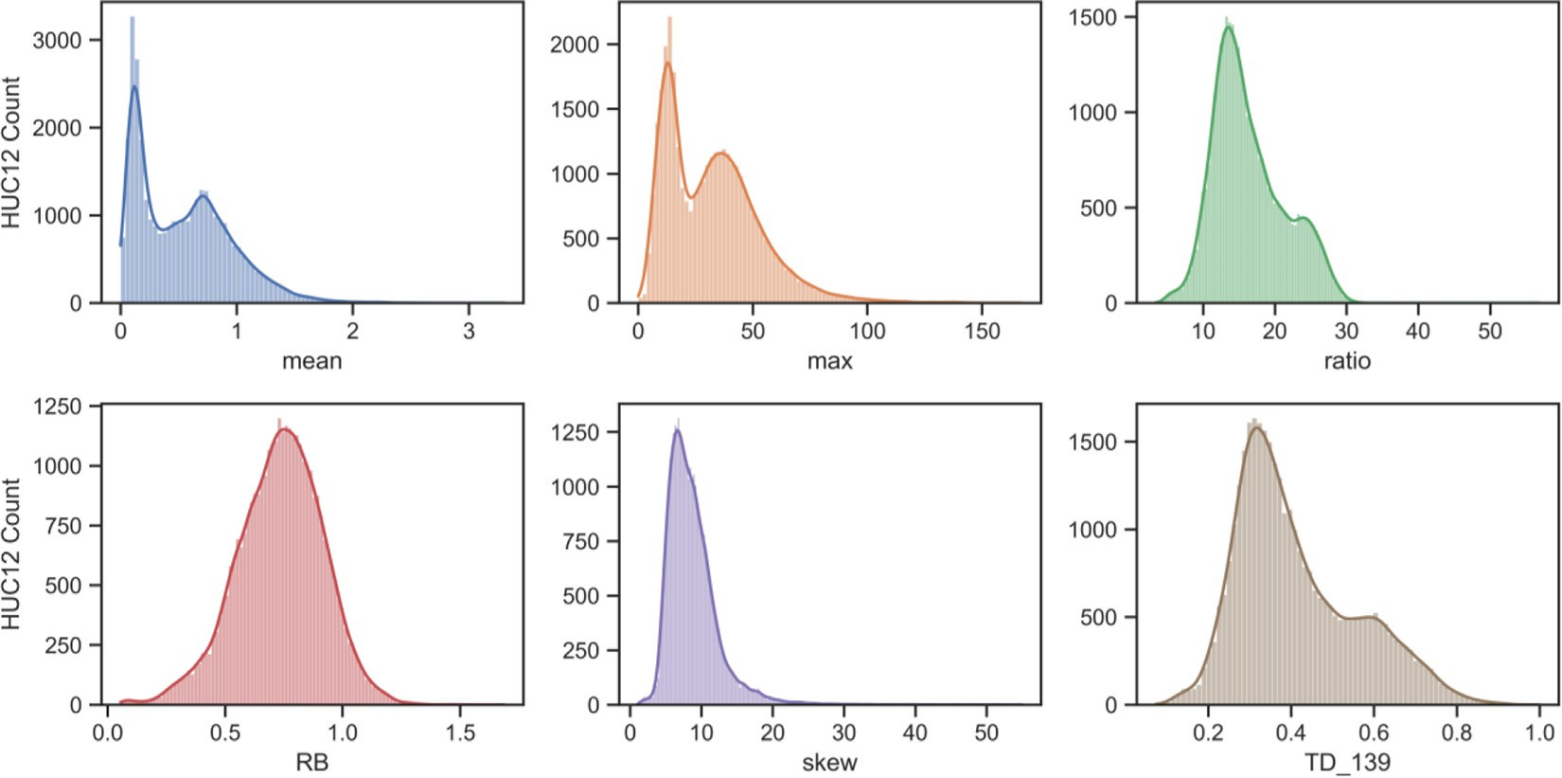
Histograms for various flood metrics calculated for the 34,897 HUC12 watersheds in the MARB.

A histogram of mean streamflows for the ~35,000 HUC12s showed a clear bimodal distribution, with more than 3000 watersheds plotting in a region less than 0.2 mm annual flow and a much wider distribution of HUC12s around a central value of approximately 0.8 mm (Fig 3). The distribution of maximum flows was also bimodal, but the distribution of flows associated with the RB index and skewness were more normally distributed (Fig 3). Bimodality was less evident in the ratio and TD metrics.

### Spatial patterns in the MARB

**Fig 4:**
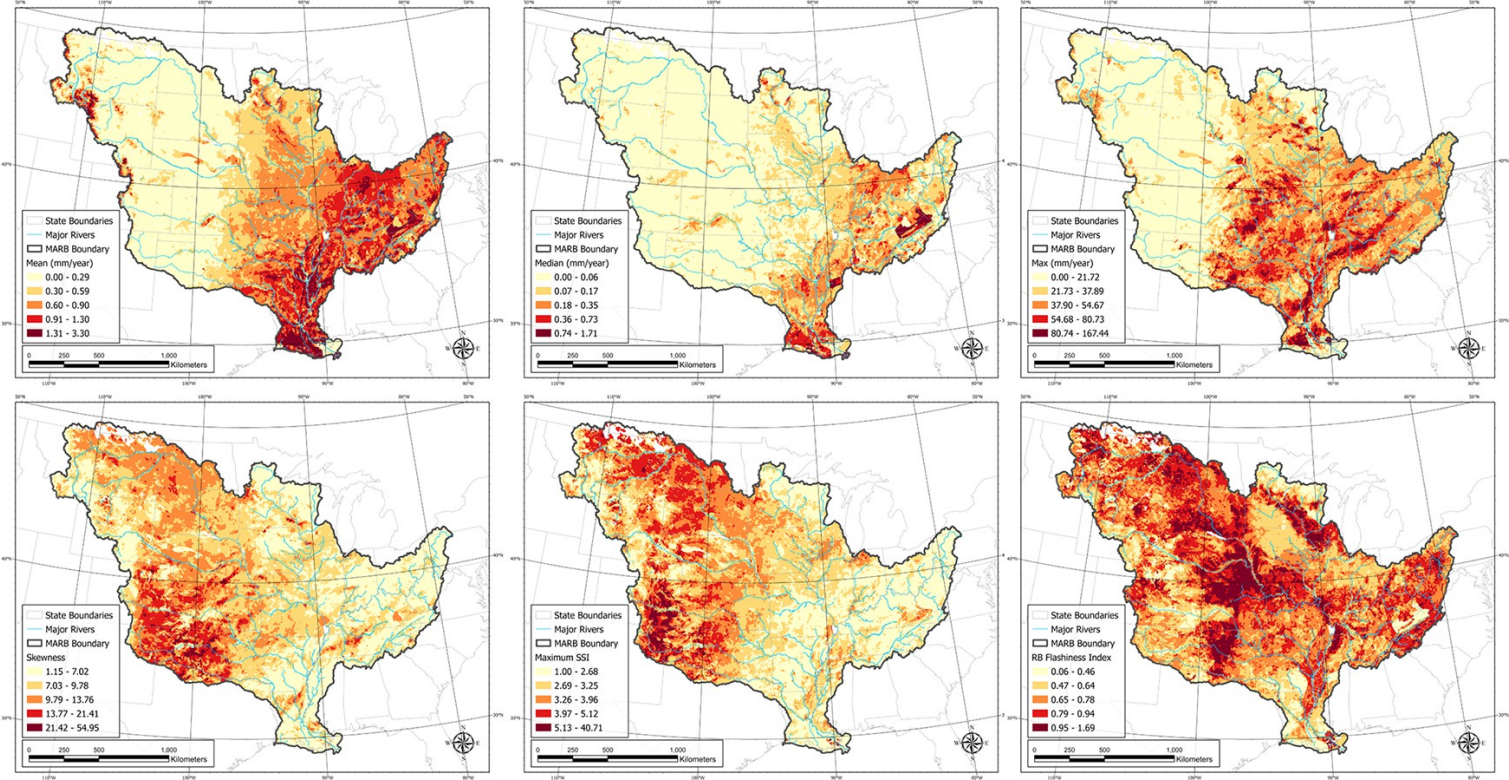
Maps of the HUC12 subwatersheds within the MARB color-coded by various flood metrics: A) mean, B) median, C) maximum daily flow, D) skewness, E) maximum SSI, and F) RB flashiness index.

Spatial patterns of streamflow metrics evaluated in this study varied among the HUC12 watersheds within the MARB (Fig 4). Watersheds with higher mean flows were primarily located in the eastern half of the MARB (Fig 4A). Similarly, higher median and maximum daily flows were situated in the southeast and eastern portions of the MARB, respectively (Fig 4B and 4C, respectively). Watersheds with the highest 90^th^ and 99^th^ percentile flows were likewise located in the eastern portion of the MARB. In contrast, streamflow values associated with metrics such as skewness, SSI, and various TD classifications were much higher in western MARB watersheds (Fig 4C, 4D, and S1 File). The RB index and ratio metric did not show obvious regional patterns, with higher values more evenly distributed in HUC12s across the MARB (Fig 4F).

**Fig 5:**
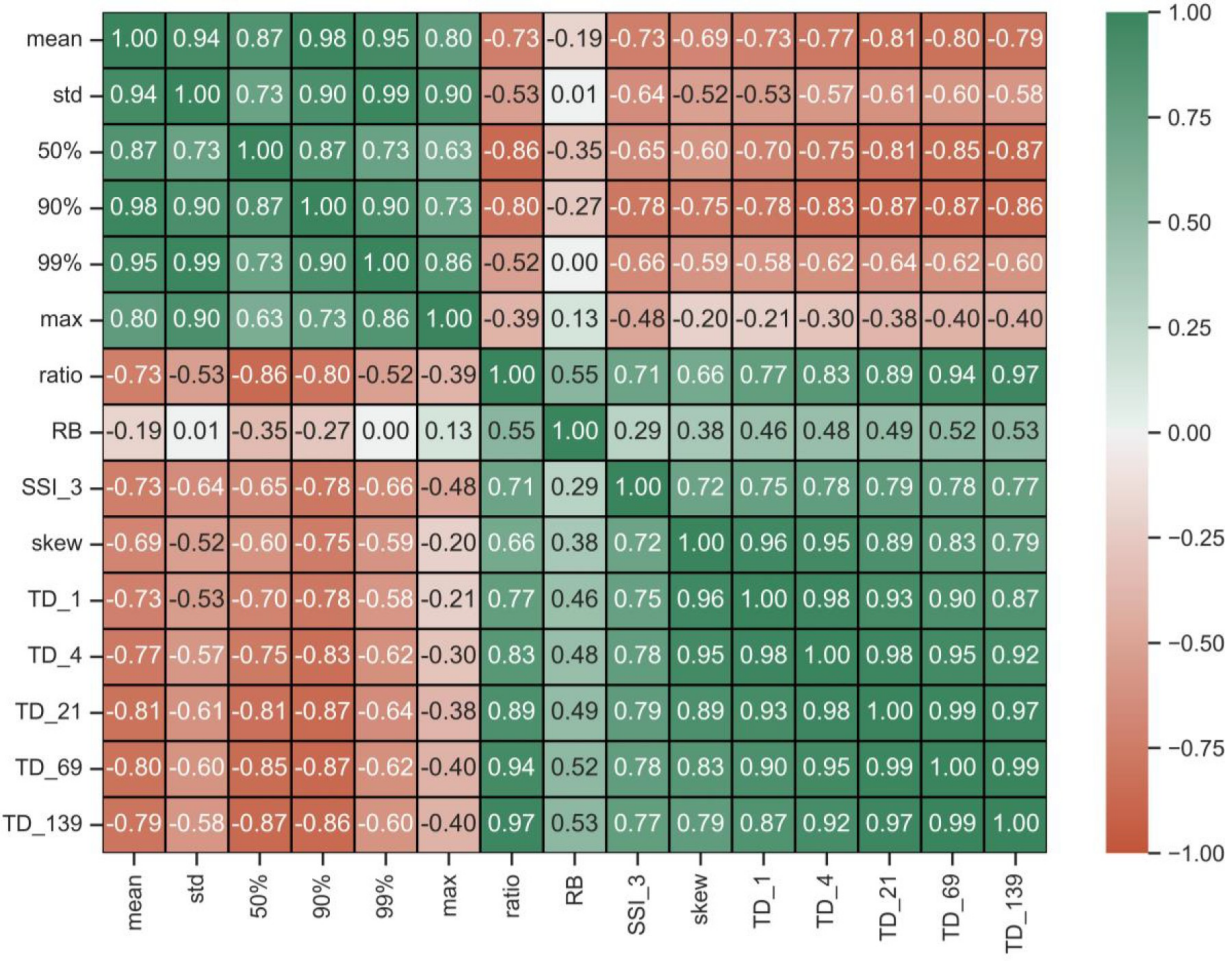
Matrix containing the Spearman correlation coefficients among various flood metrics.

The boundary between the eastern and western regions follows a line of longitude of approximately 100°W (Fig 1). East of this line, streamflow values were higher for metrics describing greater overall flows (mean, median, standard deviation, and percentiles) whereas west of the 100°W line, streamflow metrics favoring extreme values were highest (SSI, skewness, TD). A correlation matrix among the various streamflow metrics supports these spatial patterns (Fig 5). Spearman correlation coefficients of HUC12 streamflow values were highly positively correlated among the mean, standard deviation, median, and percentile rankings (r = 0.63–0.99). Similar positive correlations were observed among the SSI, skewness, and top day metrics (r = 0.72–0.99). However, the two general classes of streamflow metrics were negatively correlated with each other (Fig 5). The RB index was not well correlated with higher average flows (i.e., means) but had a higher degree of correlation with extreme flow metrics (i.e., SSI, TD).

### Flood damages

Expected annual losses (EAL) from flooding including damages to buildings and agricultural land were aggregated at the HUC12 level for the MARB. Monetary losses for buildings greatly exceeded expected annual losses for agricultural lands (Table 2). In watersheds where damages were reported, mean annual losses for agricultural lands ($3,815) were substantially lower than mean annual damages reported for buildings ($25,232), but the standard deviations associated with the losses were very high for both. Indeed, the minimum reported damage was $0, and there were no damages reported up to the 25^th^ percentile of HUC12 watersheds, and only $31 and $419 of losses were reported for agricultural lands and buildings, respectively, for the 50^th^ percentile (Table 2). EAL were highly skewed for both damage measures, with maximum building losses exceeding $16 million for one HUC12 watershed. Because building losses were so much greater than agricultural losses, their behavior dominated total annual losses.

**Table 2:** Descriptive statistics for annual estimated losses due to flooding in all HUC12 subwatersheds within the MARB.

stat	Agricultural	Building	Total
count	34,897	34,897	34,897
mean	3,815	25,232	28,911
std	13,610	293,166	293,579
min	0	0	0
10%	0	0	0
25%	0	0	23
50%	31	419	1,339
75%	1,711	4,946	10,865
90%	9,830	26,529	39,668
99%	57,745	347,362	359,334
max	541,707	16,193,943	16,194,027
sum	133,116,100	880,532,700	1,008,918,000

**Fig 6:**
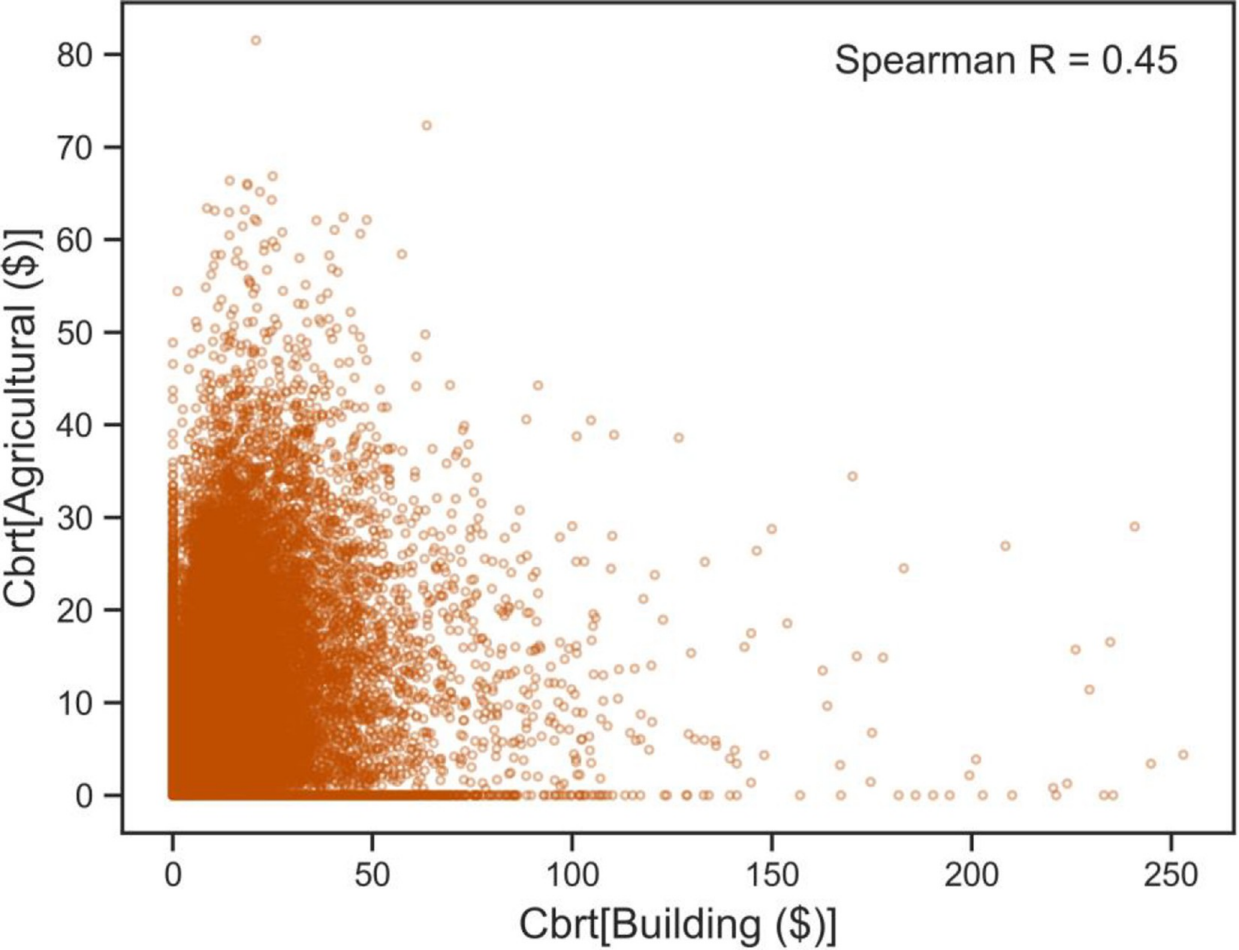
Scatterplot showing the relation between agricultural losses and building losses. Both datasets have been transformed using cubic roots.

EAL for agricultural lands and buildings were weakly correlated within the population of HUC12 watersheds of the MARB (Spearman correlation coefficient of 0.45; Fig 6). A cubic root transformation for both parameters was needed to account for the high degree of skewness and high number of zero values in the datasets.

### Relation of flood damages to flood metrics

**Fig 7:**
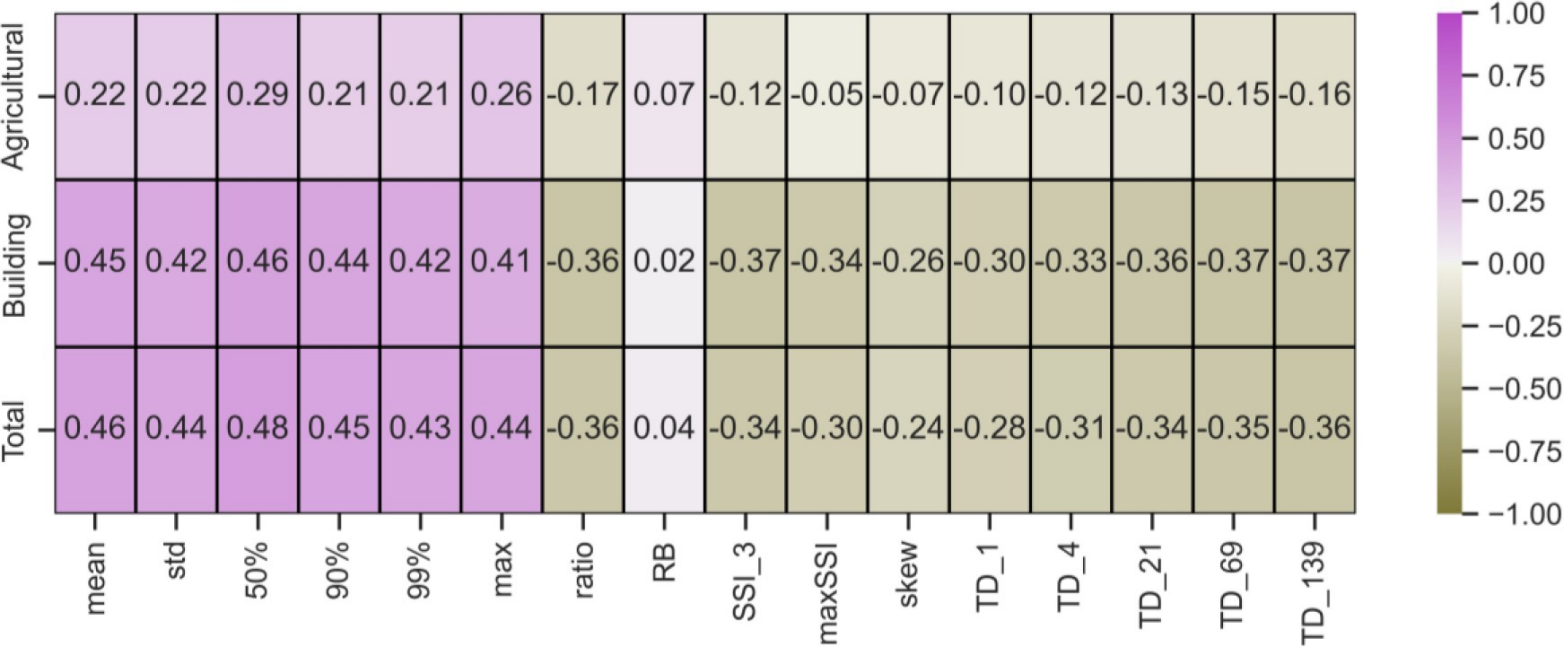
Matrix containing the Spearman correlation coefficients between losses due to flooding and various flood metrics.

Total agricultural and building losses within HUC12 watersheds were related to their flood streamflow metrics (Fig 7). Agricultural and building damages were positively correlated to the streamflow metrics describing greater overall flows (mean, median, etc.) and negatively correlated to metrics describing extreme values (SSI, TD). A higher degree of positive correlation was associated with building losses (r = 0.41–0.46) than agricultural losses (r = 0.21–0.26), and the higher building loss correlation was largely responsible for the overall correlation between total economic losses and the streamflow metrics describing higher overall flows (r = 0.43–0.48).

**Fig 8:**
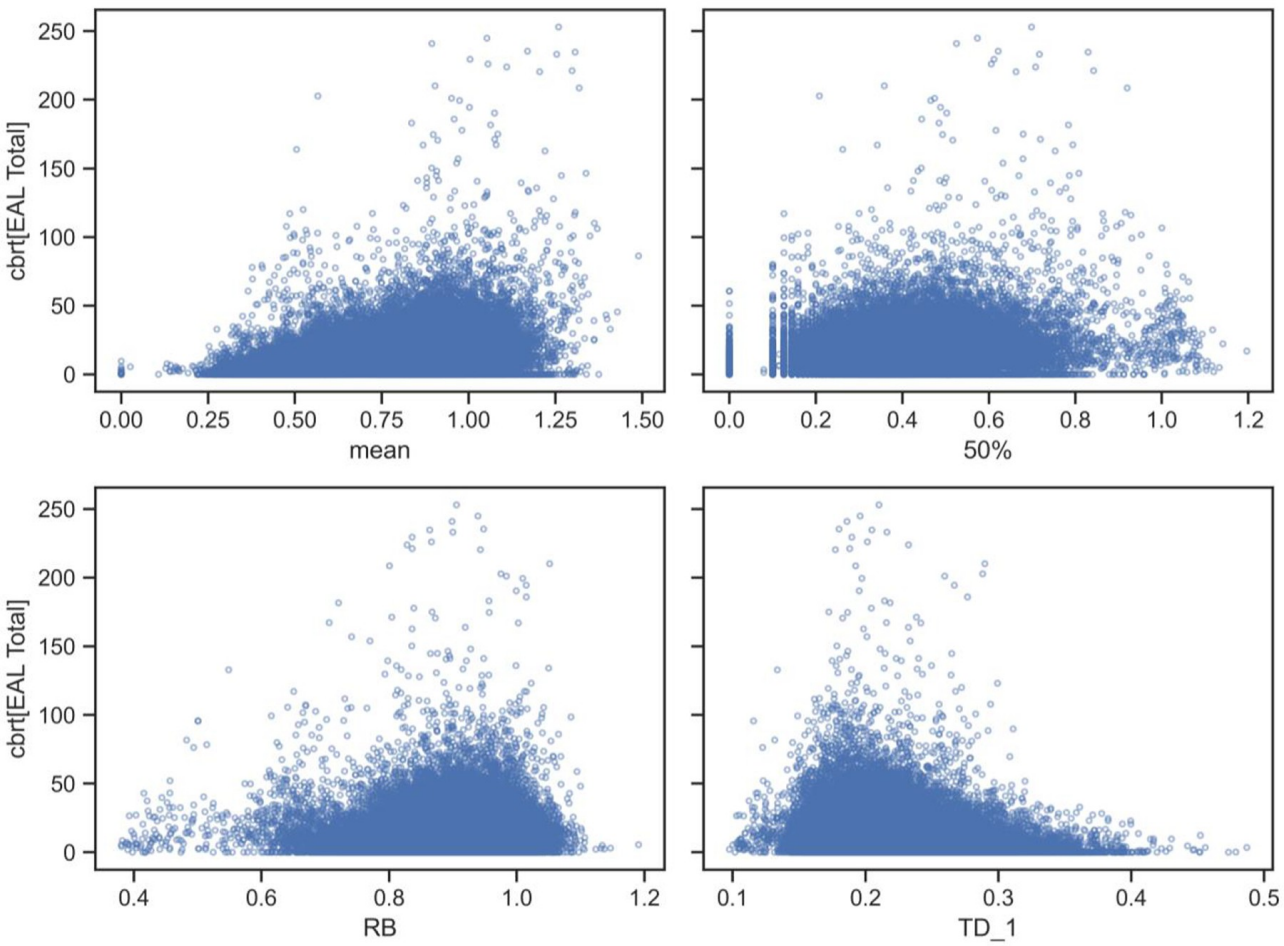
Scatterplots between total flood losses and various metrics.

On the other hand, economic losses were negatively correlated with streamflow metrics describing extreme flows (Fig 7). This should not be interpreted to imply that extreme flows do not result in damages but rather that within the HUC12 watersheds of the MARB, areas with greater risk to extreme flows have lower numbers of agricultural lands and buildings reported to be damaged by flooding. The overall positive and negative correlations of flood metrics and total EAL are shown in Fig 8 for the mean and TD metrics. A general positive increase in flood damages with increasing mean annual streamflow was evident, whereas a negative slope was observed in the relation of the TD1 metric to total damages. For both plots, it is important to note that there is a lot of scatter in the data but the plots serve to highlight a general pattern across the thousands of HUC12 watersheds of the MARB.

## Discussion

The focus of this study was to identify streamflow metrics that best correlate with reported flood damages within the ~35,000 HUC12 watersheds of the MARB. Our novel approach is substantially different than other flood-risk studies that utilize measured data at a single site (e.g., [14, 15]), develop estimates of flooding risks at ungauged sites (e.g., [24, 25]), evaluate spatial flood damages [28] or consider other hydrologic parameters as a proxy for risk [32, 33]. Within the 3,208,700 km^2^ MARB, where climate, topography, soil, hydrology, and land cover conditions are highly variable, flood risks are not equally distributed among the thousands of sub-watersheds in the basin. Using watershed-scale, model-generated daily streamflow data from a national hydrologic model for a 19-year period, we calculated 16 different streamflow metrics that could be used to assess flood risks across the basin and then correlated these metrics with measured flood damages.

### Comparison of flood metrics

Hydrologic results showed a clear spatial difference within the MARB among two different classes of metrics, those describing higher overall flows such as the mean, median, and percentiles, and those characterizing extreme flows such as SSI, skewness, and TD. The 100°W line of longitude provides an approximate line of demarcation between the two flood-metric types (see Fig 4). In the eastern half of the MARB, greater annual precipitation leads to greater mean and median annual streamflows and greater flows associated with various statistical percentiles (i.e., 90^th^, 99^th^, maximum). Flooding is common in the Mississippi and Ohio River basins (e.g., [80–82] and is often driven by climate perturbations [8, 83]. Among these “normal-expected” flood metrics, there was a high degree of correlation, ranging from 0.63 to 0.94 (Fig 4), indicating these metrics were largely reporting on the same statistical pattern. The mean flow metric had the highest degree of correlation with other “normal-expected” variables.

In contrast, west of the 100°W line, conditions in the High Plains and Mountain West are considerably drier, and average daily streamflows are considerably lower. Despite dry conditions, sudden severe storms, rapid snowmelt, and other hydrologic events in mountainous areas can trigger extreme runoff in the western region and generate streamflows that greatly exceed normal conditions. Extreme flooding has been observed in many western locations (e.g., Big Thompson and Rapid City flash floods; [84]; Platte River flooding; [85]).

Metrics describing these rarer floods include streamflow skewness and SSI, a metric that describes the number of standard deviations from the mean for a given flow value [67]. We also introduced a new metric to capture rare and extreme floods that we called “top days.” With this metric, we quantified the percentage of total discharge occurring in the wettest days associated with the 100, 99.9, 99.5, 99, and 98 flow percentiles over the period of interest (in this case, 6940 days). Although spatial patterns of 1- and 4-day metrics are similar to those shown by skewness and SSI, the addition of more days within the various TD classifications gradually extends the distribution of higher values to the east as more water is included in wetter areas (see S1 File). However, in general, the streamflow metrics describing rare but extreme floods appear to produce similar spatial distribution within the MARB.

The ratio metric, defined as the ratio between the 99^th^ percentile and the mean daily flow, was highly correlated with the “rare-extreme” flood metrics (r = 0.66–0.97). This metric is typically used to approximate bankfull flow thresholds [63, 64], and the correlation of this metric with other extreme value metrics indicates that it is capturing the bankfull capacity of larger events [60–61]. The RB flashiness index was less correlated with the extreme event metrics (0.29–0.53) and was more uniformly distributed across the MARB. Further, the area of the basin highlighted by a higher RB index was unlike other spatial patterns (favoring the lower high plains and lower Mississippi areas) and thus was not particularly useful in our analysis.

The spatial overlap of flood metrics reveals that many of them broadly describe the same type of hydrologic behavior. Most statistics evaluated in this study seem well-categorized using two spatial groups: “normal-expected” and “rare-extreme.” Using multiple metrics within a group to identify flood risk would likely yield similar results due to the co-linearity of many values in subwatersheds. Studies exploring hydrologic indices have also found intercorrelation [86, 87] and noted that the resulting statistical redundancy might lead to ineffective decision-making when applying related metrics simultaneously [88].

### Flood metrics and flood risk

Among the flood metrics evaluated in this study, those focused on higher overall flows were more correlated to EAL than the extreme value metrics (Fig 7). Although the correlation with agricultural land flood losses was not particularly strong (0.21–0.26), the correlation to building losses was higher (0.41–0.46), and the overall correlation was 0.43–0.48. In contrast, the extreme value metrics were more weakly, negatively correlated to EAL.

The better correlation of EAL to higher overall “mean” flows is consistent with geographic patterns of human settlement and agricultural production occurring in the eastern portion of the MARB (east of 100°W). Human societies evolved to be alongside rivers [89] and towns, cities, and larger metropolitan areas adjacent to rivers are prone to flooding and building losses. Recent major riverine floods include those in eastern Iowa [90], Memphis [91], and New Orleans [92], but most river floods do not receive scientific scrutiny. Instead, flood damages are often used to track impacts [28, 30]. While building losses within the MARB are concentrated in HUC12s that include major rivers, many watersheds report few, if any, losses. Indeed, half of the HUC12 watersheds in the MARB had building losses less than $419 (Table 2). However, when building losses were reported in a few outlier watersheds, damage amounts reached upwards of $16 million. Hence, a cubic root transformation was needed to account for the abundance of zero values and the high degree of skewness in the data.

Agricultural losses were also concentrated in the eastern part of the MARB, primarily in Iowa, Illinois, Indiana, the eastern Dakotas (glaciated regions), and along the floodplains of major rivers. The Upper Midwest regions dominated by corn and soybean production are continually threatened by excess precipitation [93]. For example, nearly $2 billion in federal relief funds were distributed for the Midwest Flood of 1993 [94]. Regions with higher mean flows correlate with areas of rain-fed agriculture in the Upper Midwest and production along the floodplains of major rivers. Hence, metrics that reflect higher overall flows (mean, standard deviations, and various flow percentiles) are better correlated with agricultural damages.

Although extreme flow metrics (SSI, skewness, TD) were negatively correlated with EAL, the negative relation mainly indicates that HUC12 watersheds with greater risk to extreme flows have fewer agricultural lands and buildings damaged by flooding. Extreme floods are primarily associated with dryer and mountainous western regions (Fig 3). When extreme flooding occurs in the area, much of the floodwater is dissipated in floodplains or larger valleys where there is less infrastructure to be damaged, and the land cover is dominated by perennial vegetation. Because of this, flood metrics associated with these western areas do not represent the remainder of the MARB particularly well.

A goal of this study was to identify a metric that could be used to prioritize flood risks across ~35,000 HUC12 watersheds in the MARB where future NI practices could be installed for flood risk reductions [23]. It is important to recognize that EAL values associated with flood damages are inappropriate flood risk indicators. EAL values were based on a fixed 23-year period (1996–2019), which does not necessarily indicate future risk. A small minority of watersheds witnessed catastrophic river flooding floods during that period [90–92], resulting in a subset of HUC12s with extremely high damages. Conversely, numerous HUC12s (~25%) saw no damages from 1996–2019. The EAL values appear too prone to the random fluctuations associated with flooding to be used as a viable tool for identifying long-term risk [95].

At this point, we suggest that the mean flow metric should be used as the best indicator for flood risk. The mean flow metric correlates with building and agricultural losses occurring in HUC12 watersheds, and it represents a statistical parameter that is easily determined, well understood by users and widely comparable across the basin. Other measures of flows describing higher overall water yields, such as the median, maximum, or various flow percentiles, were similarly correlated to EAL, but the statistical differentiation and spatial distribution of these metrics were more limiting across the MARB. Unlike the median, all mean values of streamflow were nonzero. Risk identification based on the median would assign zero flood risk to a handful of watersheds, but this is prevented by using the mean. In a previous assessment of combined flood and nitrate-nitrogen risks across the MARB, flood risk was assumed to be proportional to mean flows [23]. With this study, we provided additional consideration of other flood metrics and, in the end, returned to the original assumption.

### Limitations and future work

This study was focused on evaluating flood metrics and relating them to EAL at a large continental scale and there are limitations with this approach to be acknowledged. Because most of the HUC12 watersheds in the MARB are ungauged and unmonitored, we used daily streamflow output from NAM (SWAT+ model) in our study. Although NAM remains under development and detailed model calibration and validation efforts are ongoing, the model allowed us to evaluate daily flows across ~35,000 small watersheds in the MARB. Currently there are approximately 2,850 gauging stations in the MARB and most are located on HUC8 watersheds or larger. While detailed flood analysis at these measurement sites would provide one degree of information, the scale of the watersheds and limited numbers of sites are constraining factors. Similar to the “law of large numbers” concept (e.g., [96]), we contend that the large number of watersheds evaluated in this study converge on the overall truth and believe that data quantity of modeling results at 35,000 locations outweighs the analysis of high quality data at a limited number of individual gauging sites.

The 19-year period of streamflow data available for use in this study may be sufficient to obtain robust modeling parameters [97] but that data record may not be long enough to capture the variability of flows and flood damages that could conceivably occur within the MARB. Indeed, the current NAM model was developed using the 19-year record of weather in the basin, but precipitation trends have varied considerably in the MARB over the 20^th^ century [98, 99]. Streamflow metrics considered in this study may not have captured all potential flooding in the basin in response to rare, high precipitation events that could have occurred outside of the 19-year modeling window. However, if localized flooding was potentially missed in some locations, we believe the large number of watersheds evaluated in this study are useful to identify regional patterns.

In identifying flood damages to buildings and agricultural areas, we assumed that all the flood damages to buildings occurred only in floodplains and we used the floodplain extent to apportion damages to watersheds. We do not have stage-discharge relations for the watersheds to know when an actual flood occurred and assumed that all building cells present in a floodplain could be equally affected by flooding. The EAL was based on actual reported damages, but as noted above, the 19-year window for the project period may not have been sufficient to quantify all the flood damages that have occurred in these areas. It is possible that extending the damages to a longer period of time could better identify watersheds with higher degrees of risk and relate more robustly to various flood metrics.

Despite limitations in this work, we believe the study results are useful to identify continental scale patterns of flood risks within the MARB. Future work will assess the flood metric data at state and HUC2 basin levels to identify flood risks within states and geographic regions. With this approach, there will be less variability in geography and climate to contend with and it is possible that additional resolution of flood metrics within similar regions can be explored. Furthermore, as part of the larger NI study of the MARB [23], flooding risks evaluated herein will be combined with nitrate export from the NAM at a HUC12 level to develop a risk assessment that describes both flooding and water quality risks in the MARB. From this risk assessment, future work will focus on developing a continental-scale prioritization scheme for NI practice placement to mitigate these risks [23].

## Conclusions

This study evaluated streamflow metrics within the ~35,000 HUC12 watersheds of the MARB and found that flood risks are not equally distributed among the thousands of watersheds of the basin. Using watershed-scale, model-generated daily streamflow data from a national hydrologic model for a 19-year period, we calculated 16 different streamflow metrics that could be used to describe flood risks in the basin. Mean and maximum daily flows averaged 0.54 mm and 32.3 mm among the HUC12 watersheds but varied considerably across the basin. Dimensionless values such as skewness and R-B index did not vary as much as daily flows whereas top day metrics systematically increased with a larger number of days included in the calculation. A clear bimodal distribution was observed in mean and maximum annual flows but less evident in the ratio and TD metrics. The distribution of flows associated with the RB index and skewness were more normally distributed. A clear spatial difference was observed among two different classes of metrics that was centered on the 100°W line of longitude within the Central High Plains. East of this line, flood metrics describing higher overall flows such as the mean, median, and percentiles were highlighted, whereas west of this line, metrics characterizing extreme flows such as SSI, skewness, and TD were higher.

Flood metrics focused on higher overall flows were more correlated to EAL than the extreme value metrics. Overall, the mean flow metric best correlated with building and agricultural losses and it represents a statistical parameter that is easily determined, well understood by users and widely comparable across the basin. Overall, despite challenges and limitations, we believe that results from this study are useful for identifying continental scale patterns of flood risks within the MARB and should be considered a launching point from which to improve the connections between watershed scale risks and the potential use of NI practices to reduce these risks.

## Supporting information

S1 Table SWAT models simulated daily streamflow at all HUC12 subwatersheds in the MARB from January 1, 2000, to December 31, 2018. Sixteen metrics, summarized in this table, were calculated using these time-series for each HUC12.
(DOCX)

S1 File MARB maps of flood metrics. (DOCX)
