# Correction: Evaluation of flood metrics across the Mississippi-Atchafalaya River Basin and their relation to flood damages

**DOI:** 10.1371/journal.pone.0327685

**Published:** 2025-07-02

**Authors:** Keith E. Schilling, Elliot S. Anderson, Jerry Mount, Kelly Suttles, Philip W. Gassman, Natalja Čerkasova, Michael J. White, Jeffrey G. Arnold

The sixth author's name is spelled incorrectly. The correct name is: Natalja Čerkasova. The correct citation is: Schilling KE, Anderson ES, Mount J, Suttles K, Gassman PW, Čerkasova N, et al. (2024) Evaluation of flood metrics across the Mississippi-Atchafalaya River Basin and their relation to flood damages. PLoS ONE 19(10): e0307486. https://doi.org/10.1371/journal.pone.0307486.
