# Supplementary material for: Evaluation of flood metrics across the Mississippi-Atchafalaya River Basin and their relation to flood damages
Source: PLoS One. 2024 Oct 9;19(10):e0307486. doi: 10.1371/journal.pone.0307486 (PMC11463744; doi:10.1371/journal.pone.0307486)
Supplement: S1 Table — Sixteen metrics, summarized in this table, were calculated using these time-series for each HUC12. (DOCX) [file pone.0307486.s001.docx]

Table S1. SWAT models simulated daily streamflow at all HUC12 subwatersheds in the MARB from January 1, 2000, to December 31, 2018. Sixteen metrics, summarized in this table, were calculated using these time-series for each HUC12.

| **Statistic Abbreviation** | **Formal Name** | **Unit** |
| --- | --- | --- |
| mean | Arithmetic Mean | mm |
| std | Standard Deviation | mm |
| 50% | Median | mm |
| 90% | 90th Percentile | mm |
| 99% | 99th Percentile | mm |
| max | Maximum Daily Flow | mm |
| ratio | Quotient of 99% ∕ mean | unitless |
| skew | Skewness | unitless |
| RB | Richards-Baker Flashiness Index | unitless |
| TD_1 | Top Days 1 | % |
| TD_4 | Top Days 4 | % |
| TD_21 | Top Days 21 | % |
| TD_69 | Top Days 69 | % |
| TD_139 | Top Days 139 | % |
| maxSSI | Maximum SSI value | unitless |
| SSI_3 | Number of Days with an SSI > 3 | days |
