# Supplementary material for: Evaluation of flood metrics across the Mississippi-Atchafalaya River Basin and their relation to flood damages
Source: PLoS One. 2024 Oct 9;19(10):e0307486. doi: 10.1371/journal.pone.0307486 (PMC11463744; doi:10.1371/journal.pone.0307486)
Supplement: S1 File — (DOCX) [file pone.0307486.s002.docx]

**S1Figures: Maps of metrics evaluated in this study.**


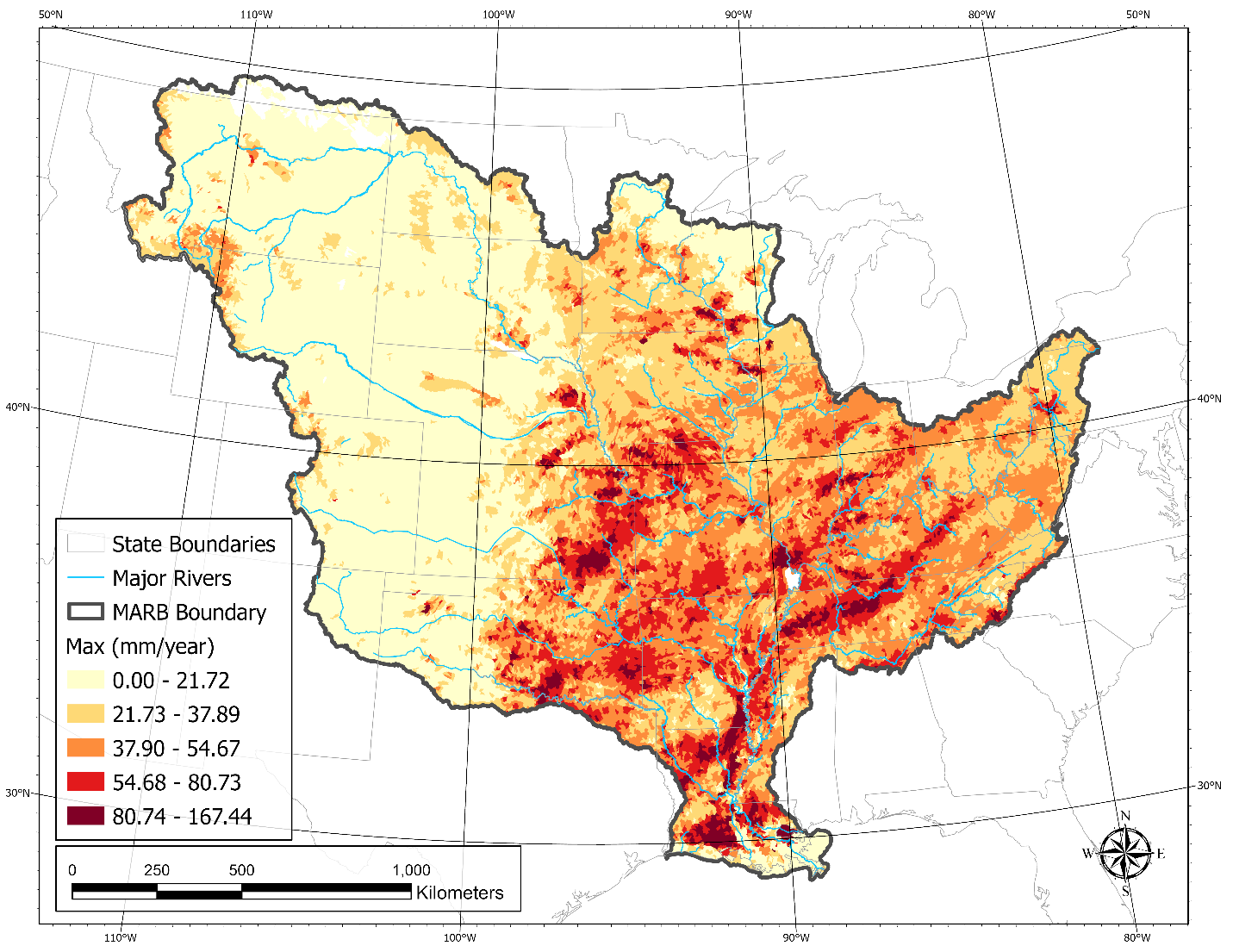


**Maximum annual streamflow**


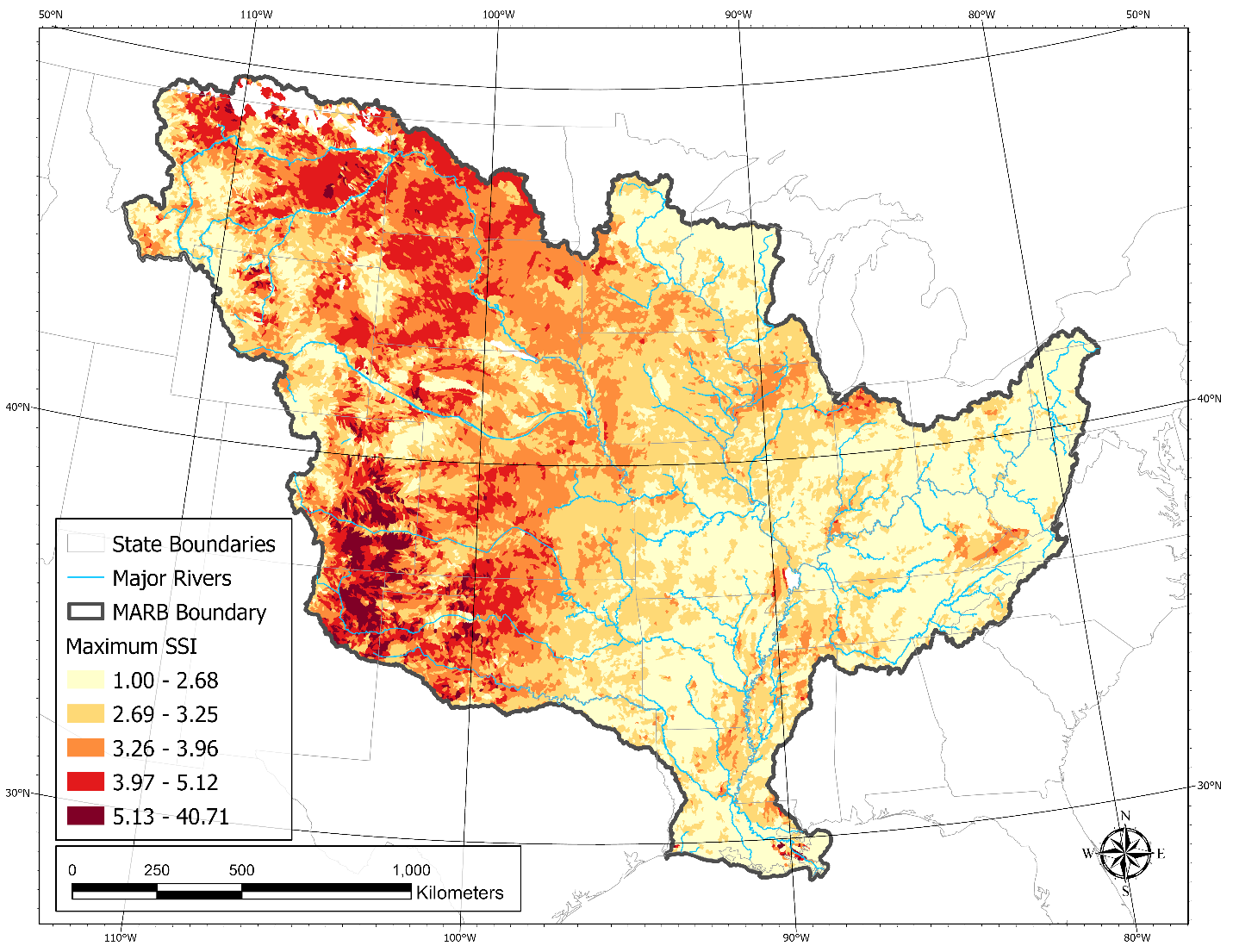


**Maximum standardized streamflow index**


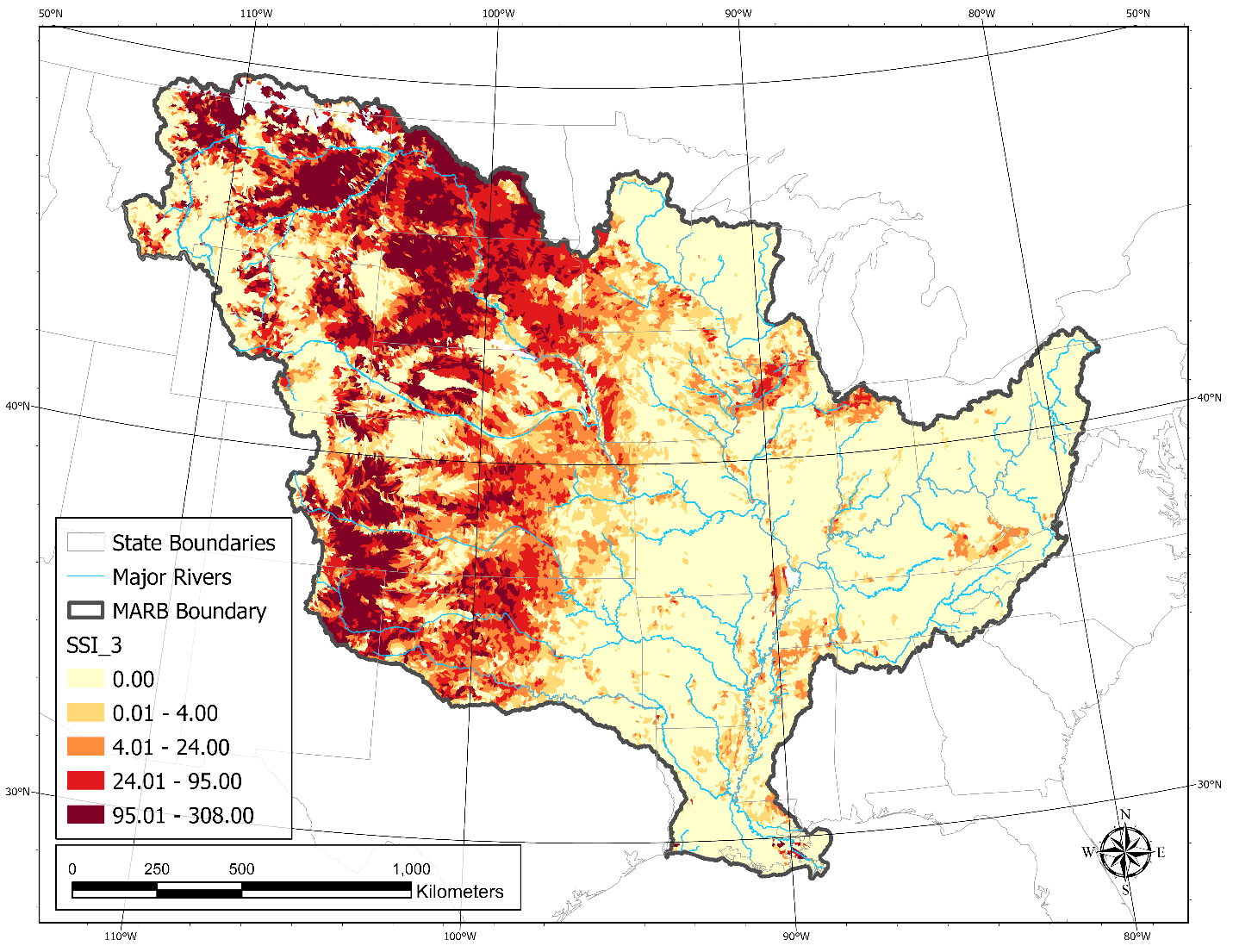


**Standardized streamflow index 3 day**


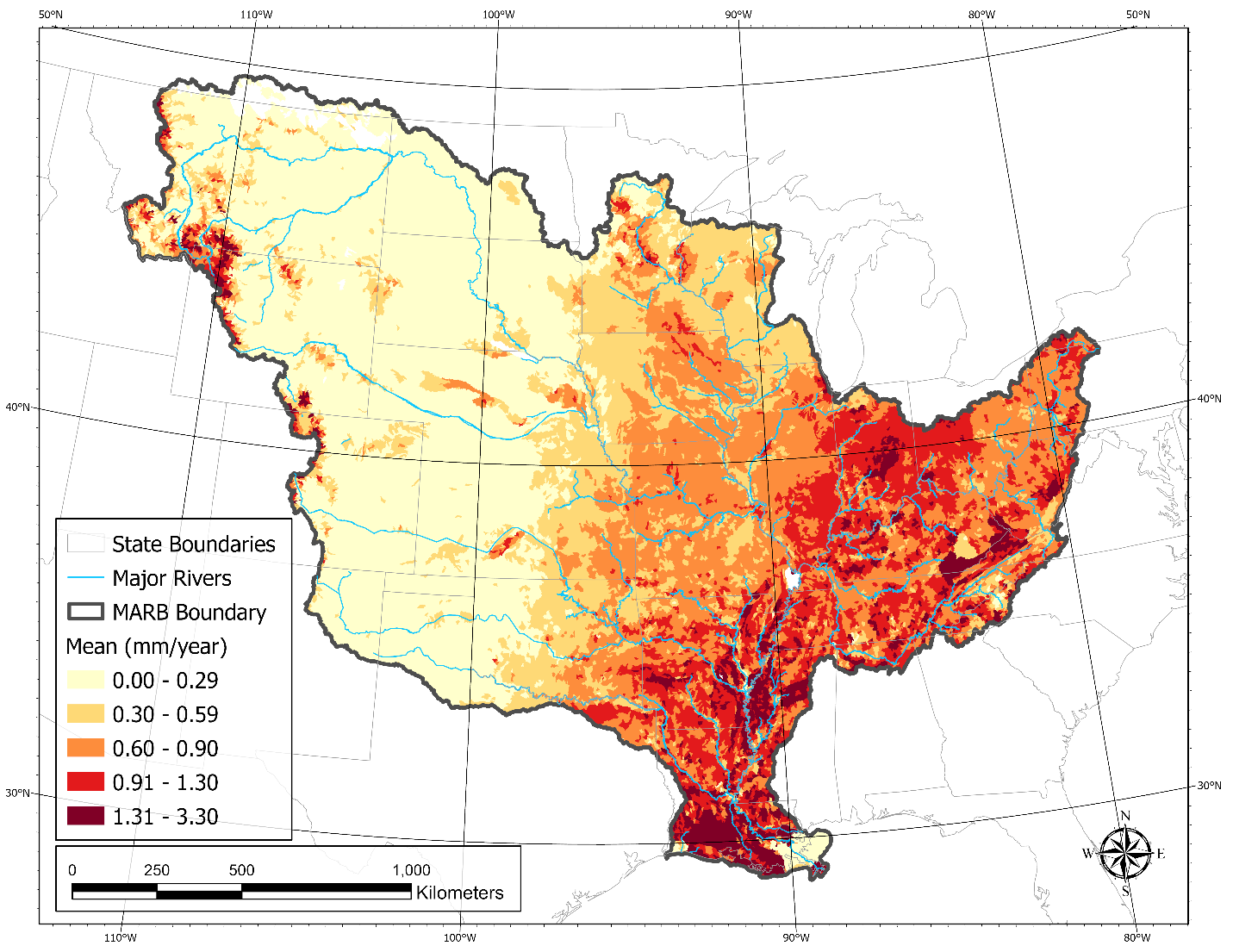


**Mean annual streamflow**


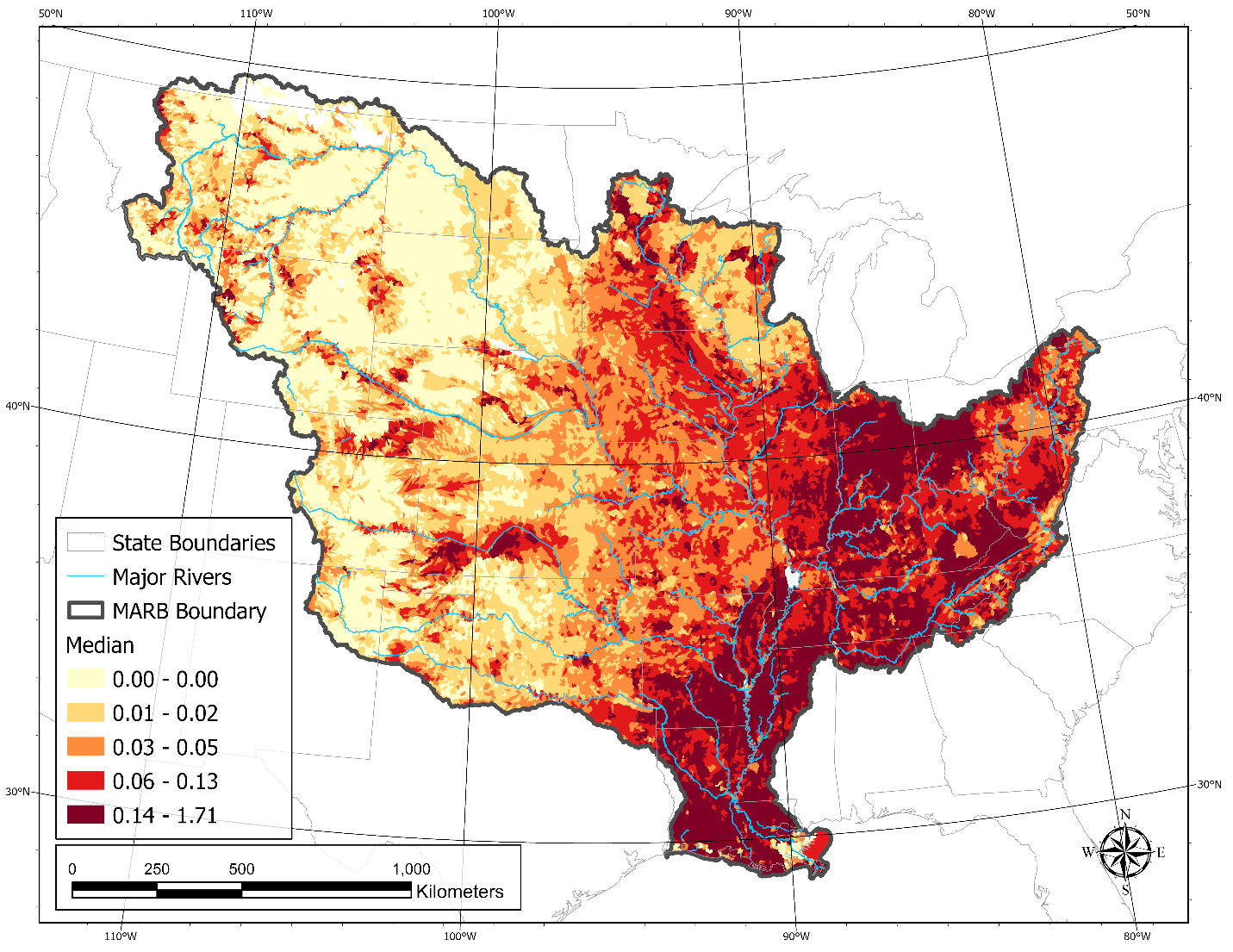


**Median annual streamflow**


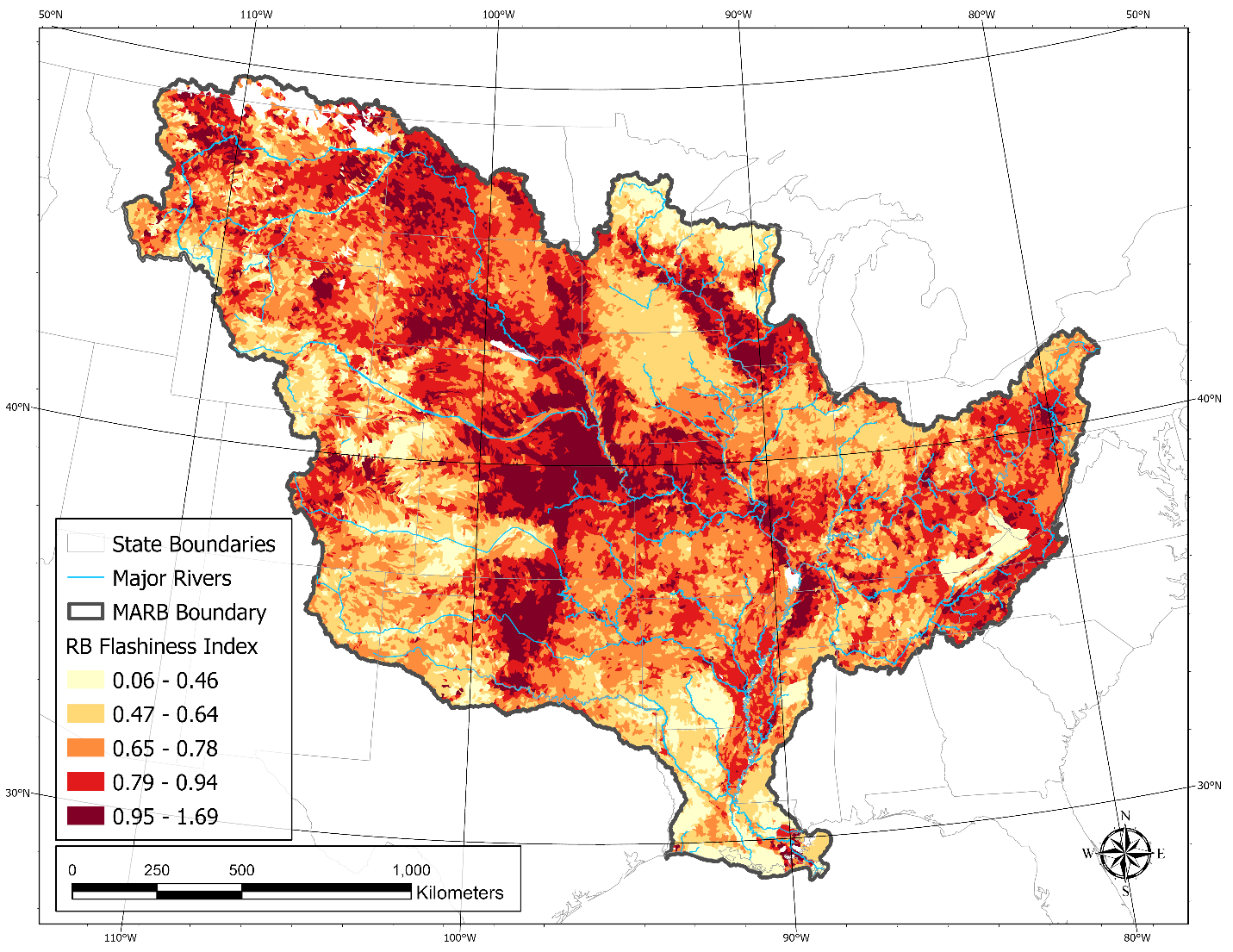


**RB flashiness index**


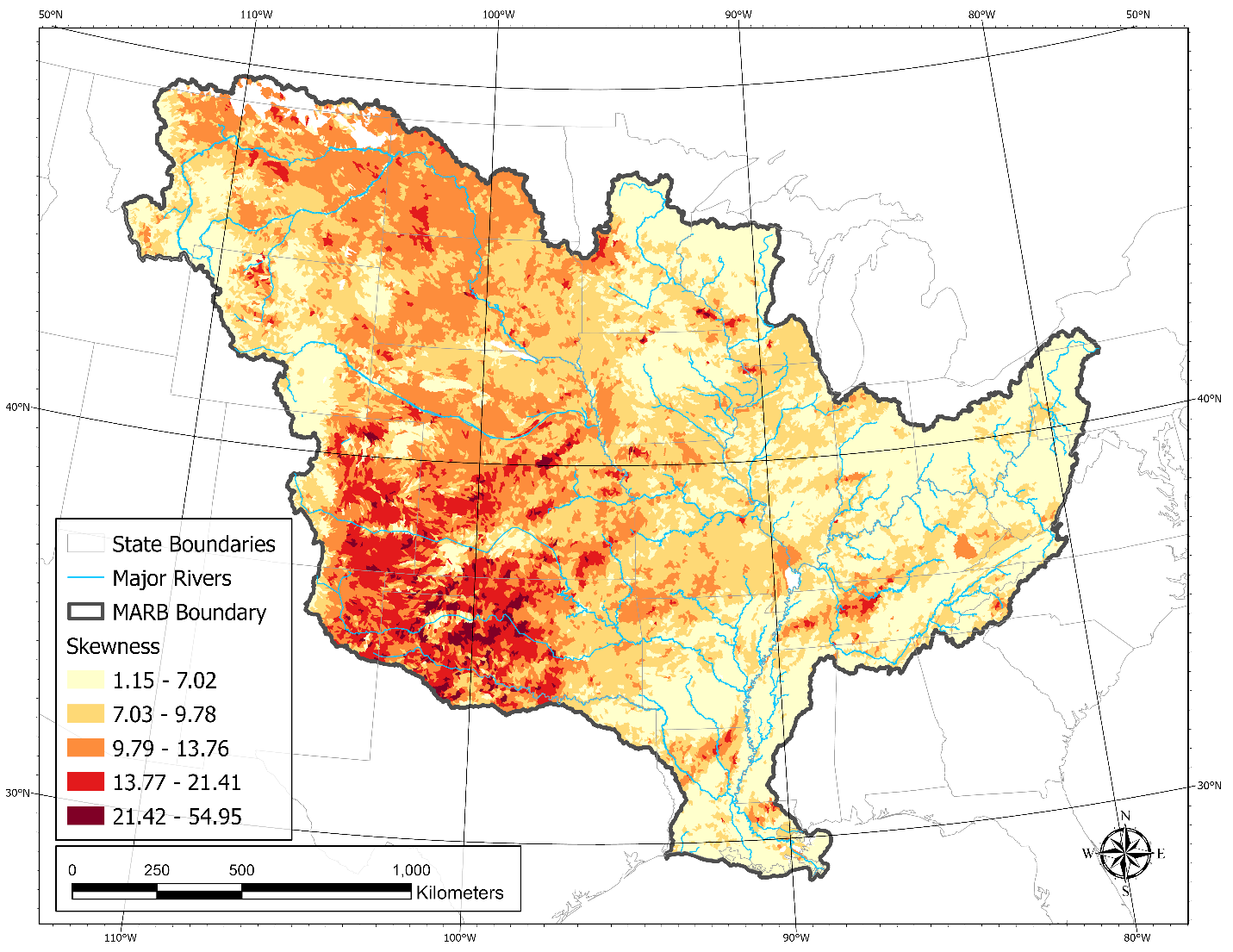


**Skewness**


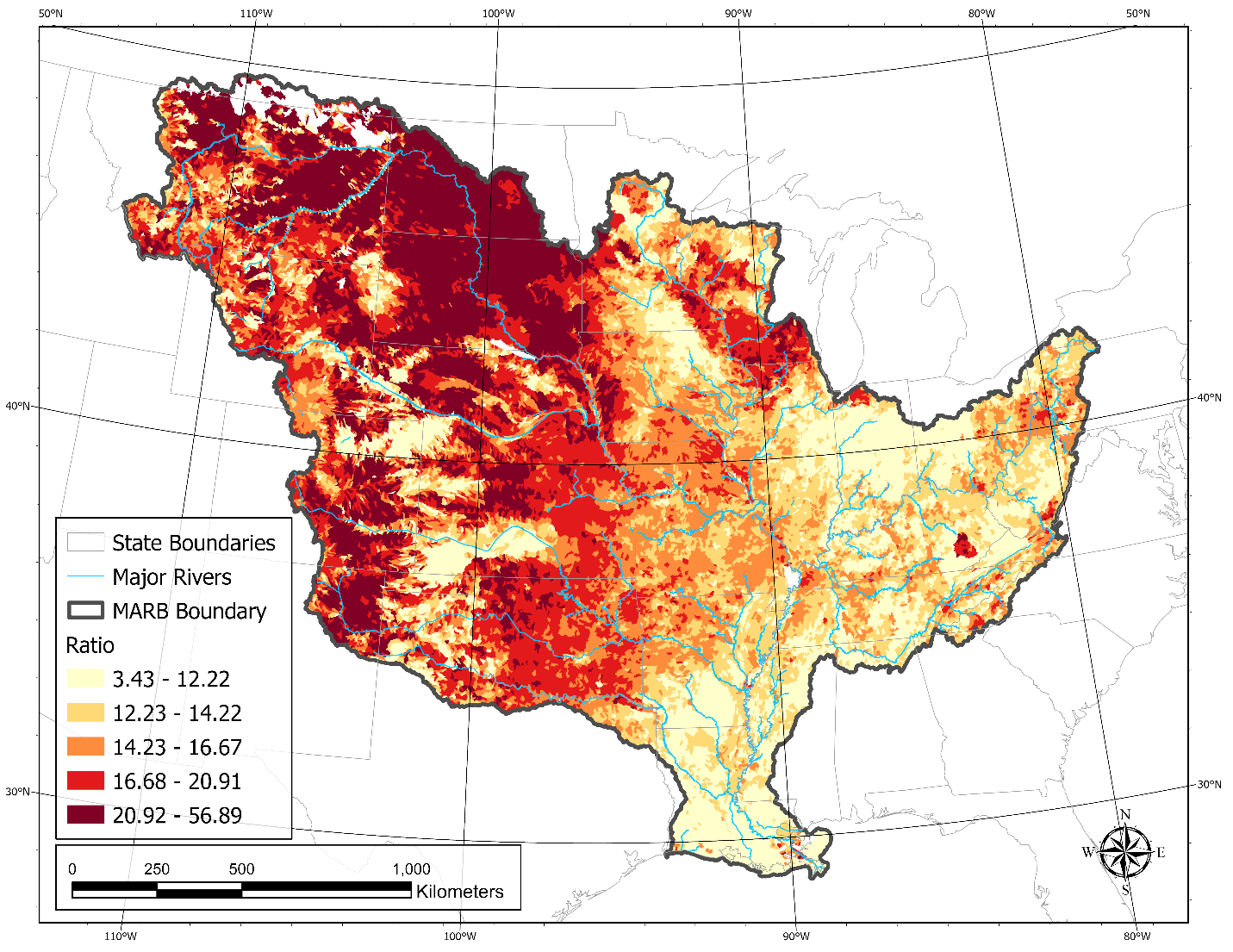


**Ratio of Quotient of 99% ∕ mean**


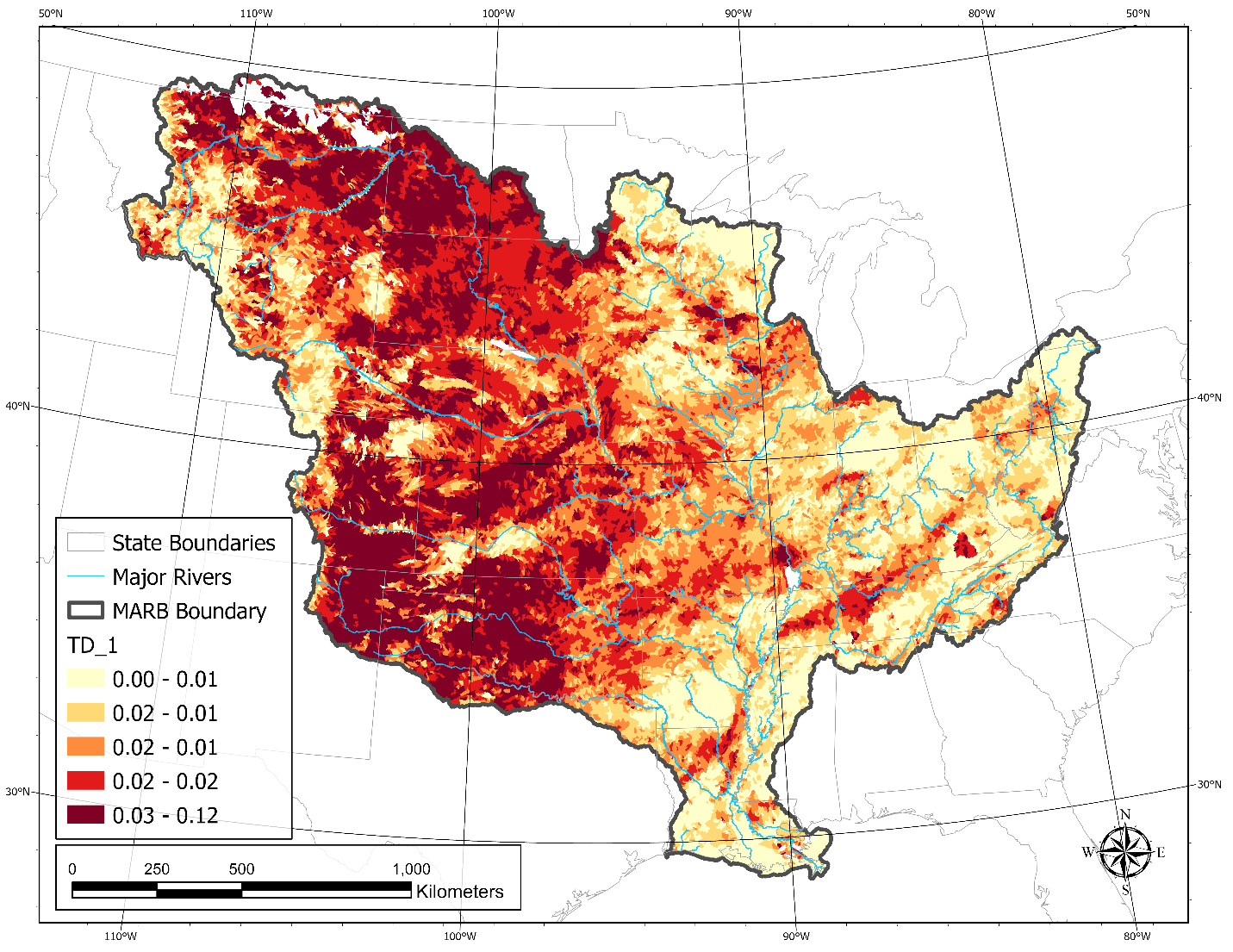


**Top days 1**


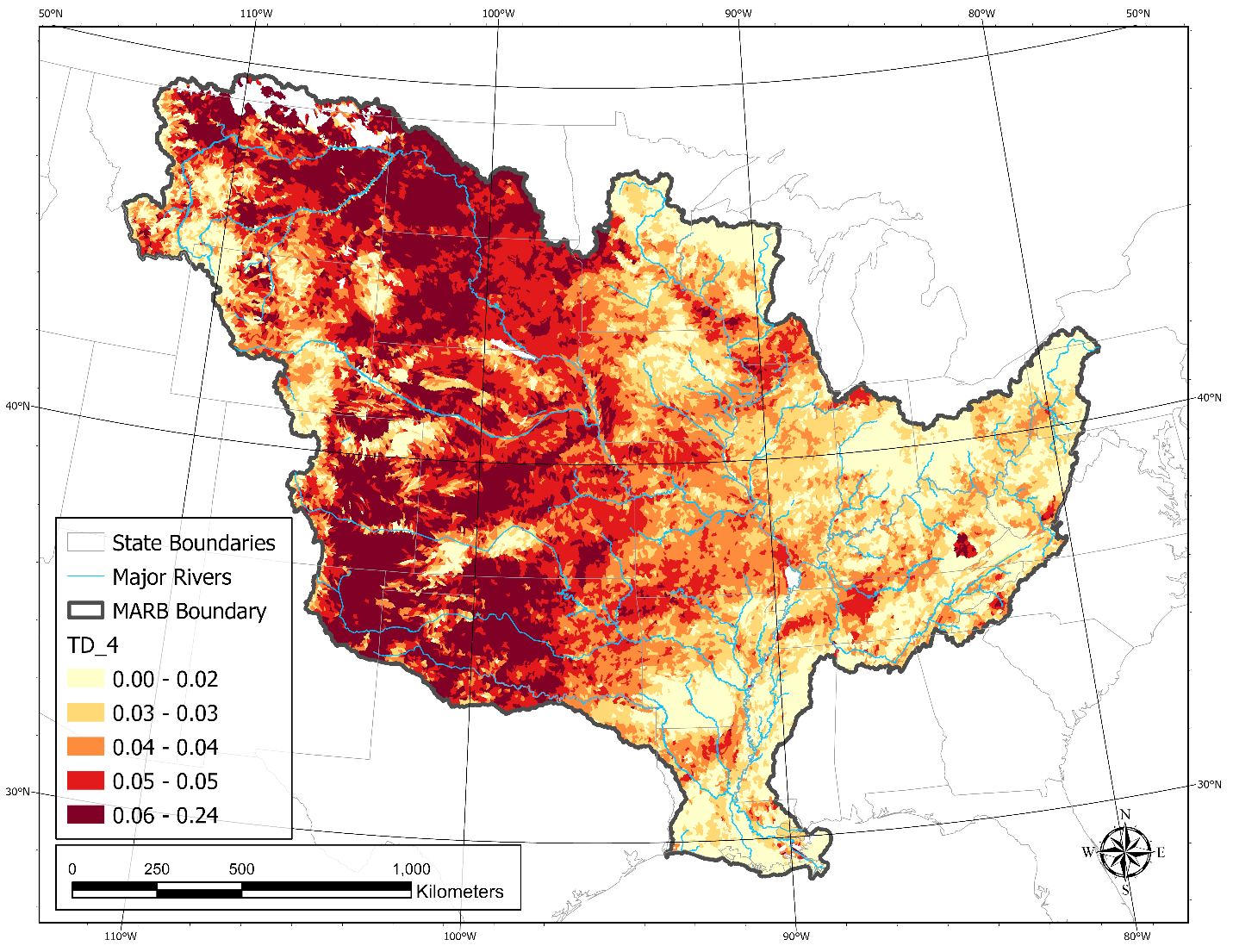


**Top days 4**


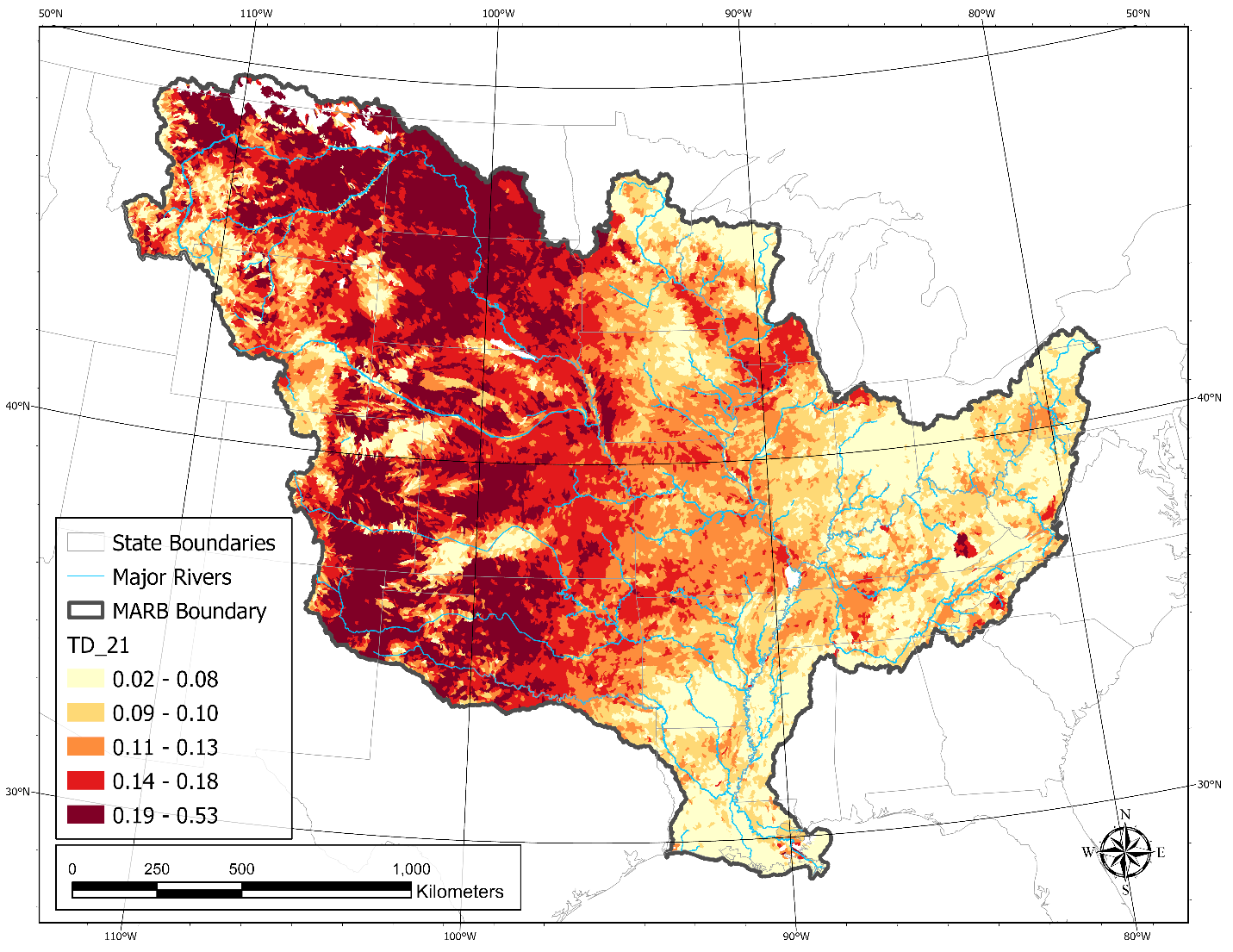


**Top days 21**


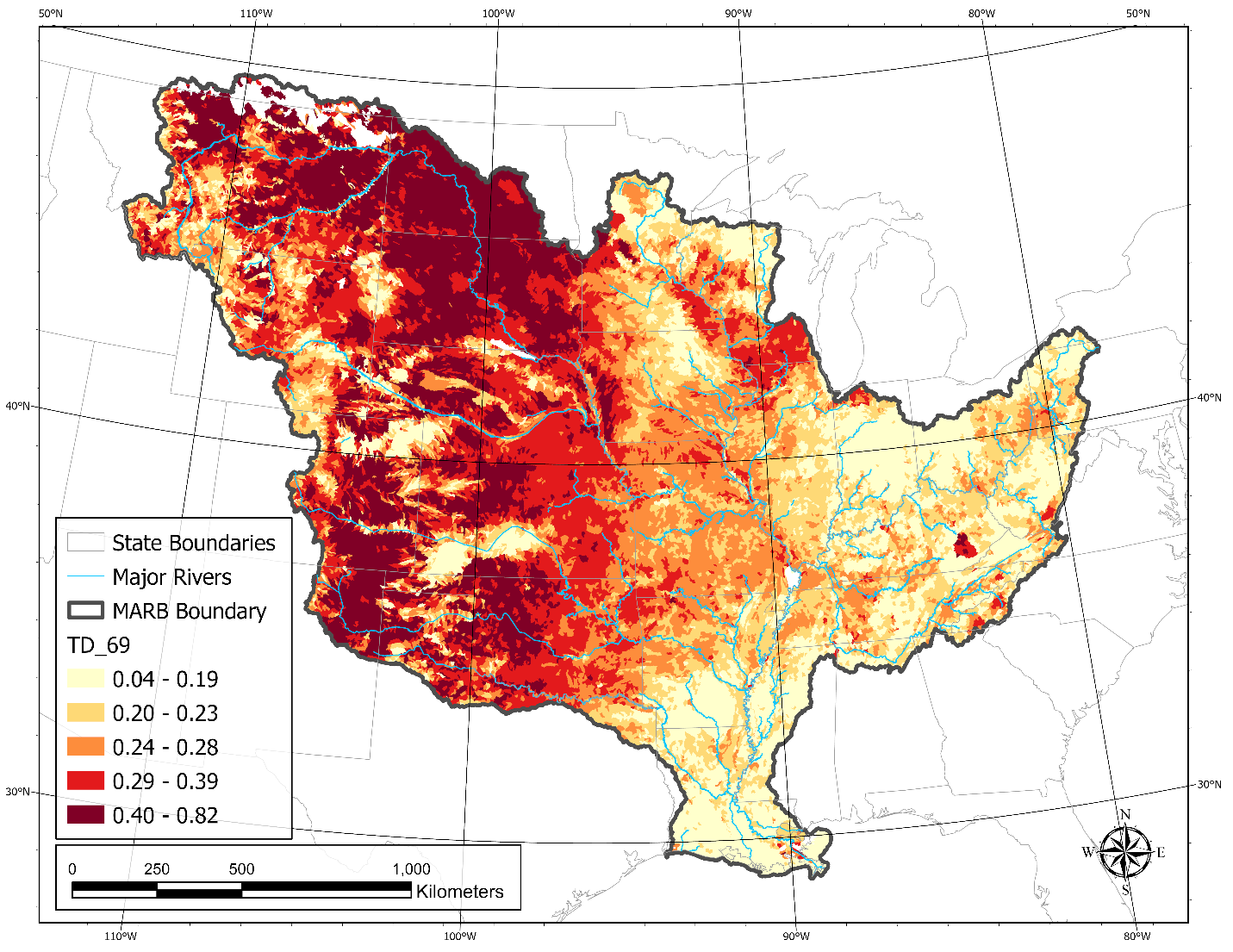


**Top days 69**


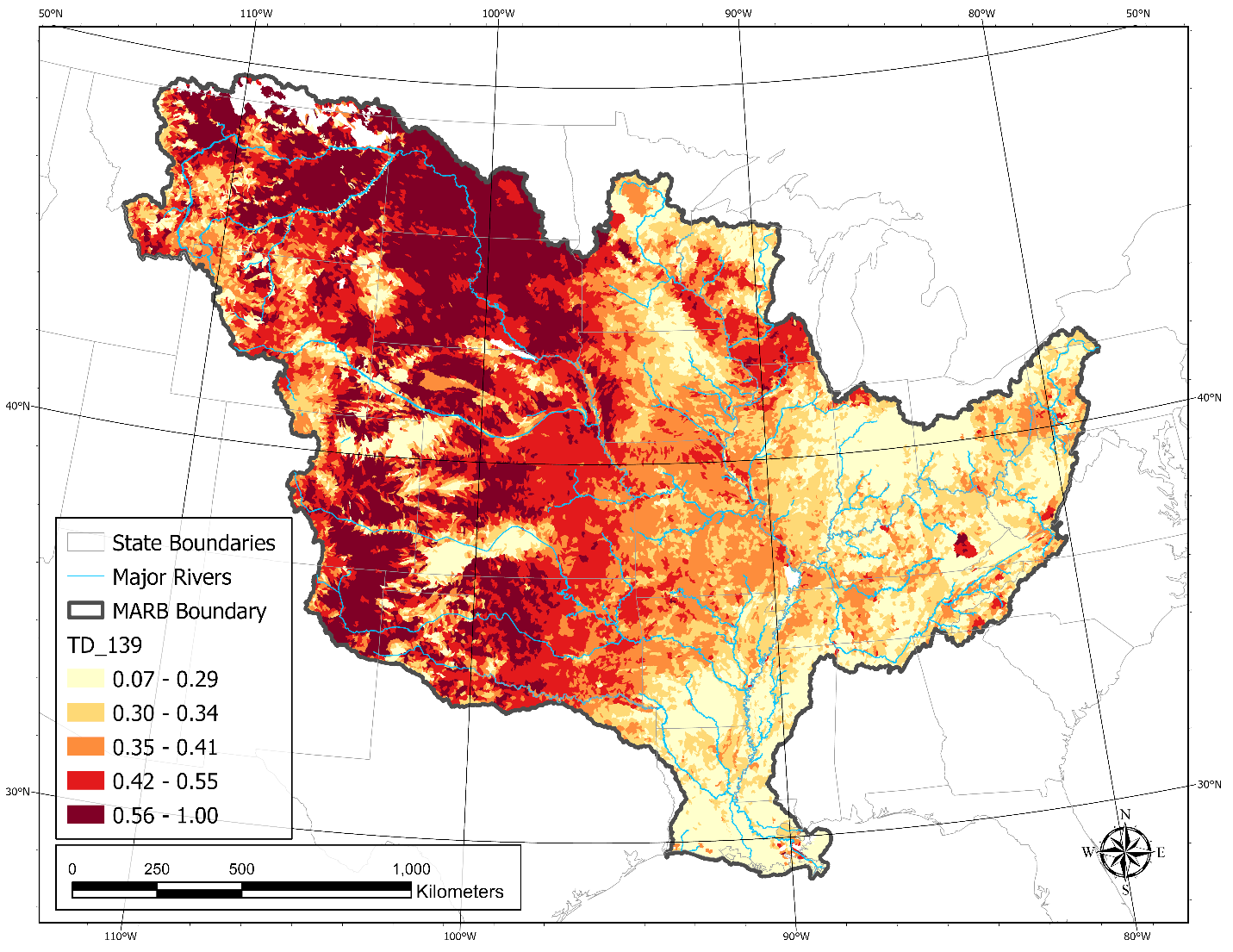


**Top days 139**


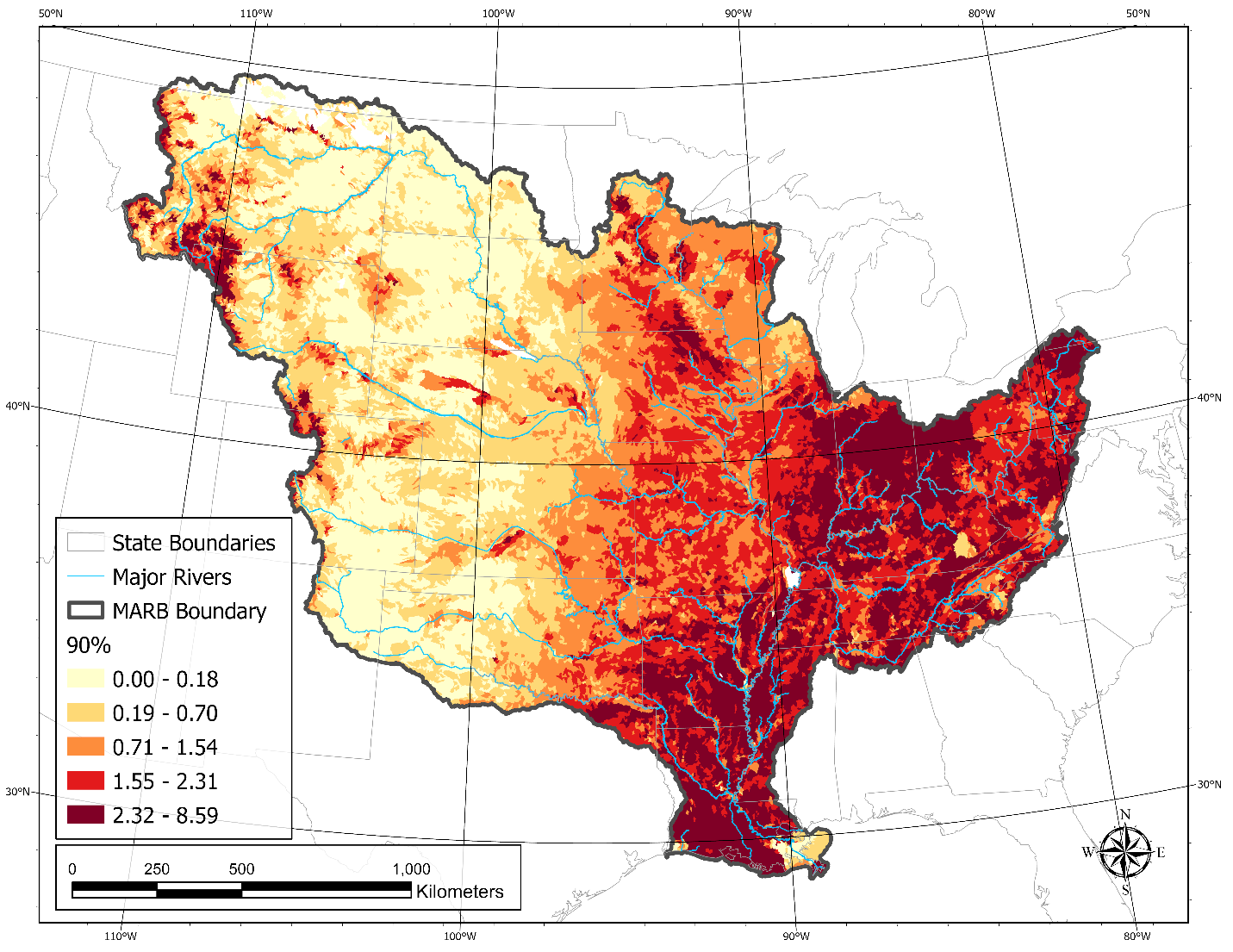


**90^th^ percentile**


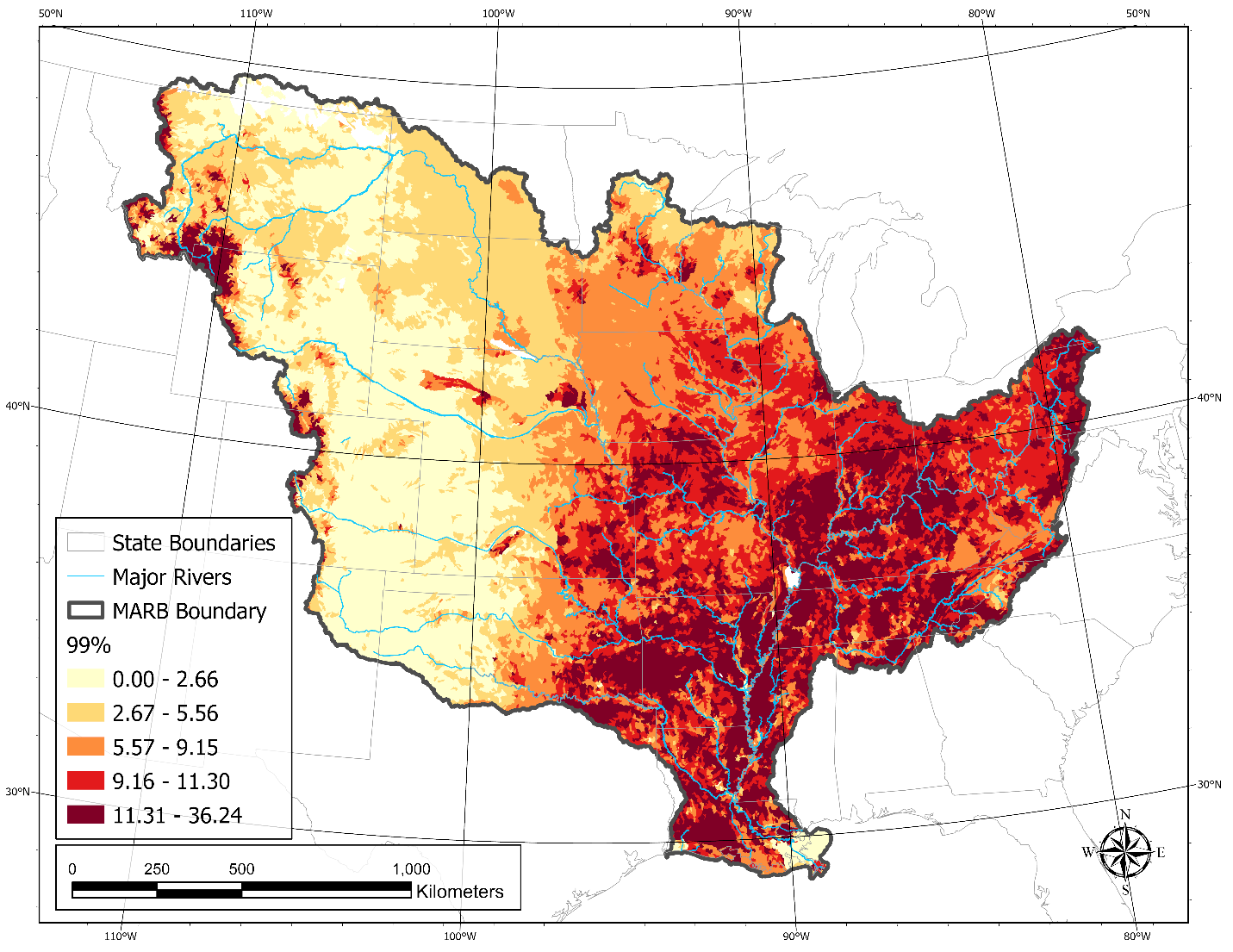


**99^th^ percentile**
